# Effective chemical protection against the maize late wilt causal agent, *Harpophora maydis*, in the field

**DOI:** 10.1371/journal.pone.0208353

**Published:** 2018-12-18

**Authors:** Ofir Degani, Shlomit Dor, Daniel Movshowitz, Eyal Fraidman, Onn Rabinovitz, Shaul Graph

**Affiliations:** 1 Tel-Hai College, Upper Galilee, Tel-Hai, Israel; 2 Migal–Galilee Research Institute, Kiryat Shmona, Israel; 3 Netafim Ltd., Tel Aviv, Israel; 4 Ministry of Agriculture and Rural Development, Consultation Service (Shaham), Beit-Dagan, Israel; Tallinn University of Technology, ESTONIA

## Abstract

Late wilt, a disease severely affecting maize fields throughout Israel, is characterized by relatively rapid wilting of maize plants before tasseling and until shortly before maturity. The disease’s causal agent is the fungus *Harpophora maydis*, a soil-borne and seed-borne pathogen, which is currently controlled using reduced sensitivity maize cultivars. In a former study, we showed that Azoxystrobin (AS) injected into a drip irrigation line assigned for each row can suppress *H*. *maydis* in the field and that AS seed coating can provide an additional layer of protection. In the present study, we examine a more cost-effective protective treatment using this fungicide with Difenoconazole mixture (AS+DC), or Fluazinam, or Fluopyram and Trifloxystrobin mixture, or Prothioconazole and Tebuconazole mixture in combined treatment of seed coating and a drip irrigation line for two coupling rows. A recently developed Real-Time PCR method revealed that protecting the plants using AS+DC seed coating alone managed to delay pathogen DNA spread in the maize tissues, in the early stages of the growth season (up to the age of 50 days from sowing), but was less effective in protecting the crops later. AS+DC seed coating combined with drip irrigation using AS+DC was the most successful treatment, and in the double-row cultivation, it reduced fungal DNA in the host tissues to near zero levels. This treatment minimized the development of wilt symptoms by 41% and recovered cob yield by a factor of 1.6 (to the level common in healthy fields). Moreover, the yield classified as A class (cob weight of more than 250 g) increased from 58% to 75% in this treatment. This successful treatment against *H*. *maydis* in Israel can now be applied in vast areas to protect sensitive maize cultivars against maize late wilt disease.

## Introduction

Late wilt, or black bundle disease, is a vascular wilt disease of *Zea mays* (corn, maize) caused by the soil-borne and seed-borne fungus, *Harpophora maydis* [1, 2]. Synonyms are *Cephalosporium maydis* (Samra, Sabet and Hingorani) and *Acremonium maydis* [2, 3]. The disease is considered to be the most harmful in commercial maize fields in Israel [4], and a major threat to corn in Egypt [5], India [6] and Spain [7]. The disease is gradually continuing to spread and is now reported in at least 11 countries (average reports since 2008 of about one new country every two years).

The primary disease symptom is a rapid wilting of maize plants, generally 70 to 80 days before tasseling and until near ripeness. The pathogen can cause damage to sprouting seeds, which results in reduced and delayed seedling emergence [6, 8]. Necrotic lesions may appear on the roots of susceptible maize plants as early as three weeks after inoculation [9], and at that time, infected seedlings have shortened roots [10]. First above-ground symptoms usually appear approximately 60 days after sowing (DAS) [11]. With disease progression, the lower stem dries out (particularly at the internodes) and has a shrunken and hollow appearance, with dark yellow to brownish softened pith and brownish-black vascular bundles [10]. Infection also results in a reduction in the number of vascular bundles in the cross-section of the internode [12].

Late wilt disease is frequently associated with infection by secondary plant parasitic fungi causing the stem symptoms to become more severe [13, 14]. Indeed, the post-flowering stalk rot complex, which includes several fungi (such as *Fusarium verticillioides* causing stalk rot, *Macrophomina phaseolina* causing charcoal rot and *H*. *maydis*), is one of the most severe, destructive and widespread groups of diseases in maize [15]. In heavily infested fields planted with sensitive maize hybrids, late wilt can cause 100% infection and total yield loss [16]. If ears are produced, the kernels that do form are poorly developed [4] and may be infested with the pathogen. More maize is produced annually than any other grain, reflecting its importance in the global market. In Israel in 2017, 2,763 metric hectares of sweet corn for the food industry were planted (5% of the total vegetable area in Israel), and 58,519 metric tons of crops (cobs) were harvested (20.12 metric tons per metric hectare). The quantity of corn crops in metric tons yield per metric hectare exhibits a constant upward tendency, from 17.5 in 1987–1996, to 18.0 in 1997–2006 and to 20.1 in 2007–2016 (data from the Israel Organization of Crops and Vegetables). Effective risk management of late wilt disease, primarily by avoiding the growth of sensitive maize cultivars, may contribute to this positive tendency. For example, the yield of the sensitive maize cultivar, Jubilee cv., dropped dramatically from a value of 18 tons per hectare in healthy fields during the years 2000–2005 to 5 tons per hectare in heavily infested fields during the years 2008–2009, together with a significant reduction in yield quality [4].

Since the increase in the incidence of late wilt disease over the past two decades (starting in the 1980s in the Hula Valley in northern Israel, an area that is now considered to be heavily infested) [4], Israeli growers are now planting resistant maize cultivars, although they often have lower market value. In fact, Israel’s leading sweet maize strain, Jubilee cv., which was the most abundant in commercial fields in the 1990s, is no longer routinely grown in Israel.

*Harpophora maydis* can survive and spread through the movement of infested soil, crop residue [17], seed-borne inoculum [1] or secondary hosts [18, 19]. Hence, the need to control this pathogen efficiently has become increasingly important in both commercial grain production and maize seed production. Indeed, some agricultural (balanced soil fertility and flood-fallowing) [2, 20], biological [21–25], physical (solar heating) [26], allelochemical [9] and chemical options [10, 27, 28] have promising potential to reduce the pathogen’s impact on commercial production. However, according to data from the Israeli Ministry of Agriculture and Rural Development (Consultation Service, Shaham), none of these methods are currently being used in Israel.

At present, late wilt disease is controlled by more economically effective management through the development of genetically resistant maize cultivars [29, 30]. The National Maize Program at the Agricultural Research Center in Giza, Egypt, identified many sources of resistance, and their release of resistant cultivars since 1980 has significantly reduced late wilt losses in Egypt [29]. A breeding program for resistant germ lines has been operational in Israel for about a decade (Israel Northern R&D, Migal–Galilee Research Institute, Kiryat Shmona, Israel, unpublished data). A recent report suggests that plant hormones may play a role in resistance to late wilt [31]. However, limited data are available in the literature on the causes of resistance in those apparently asymptomatic maize varieties.

Moreover, the presence of high aggressive isolates of *H*. *maydis* [32, 33] may threaten these resistant maize cultivars. It was also demonstrated that the pathogen could spread in relatively resistant plants that showed no symptoms [4], and that seeds of these apparently healthy and relatively resistant plants may therefore also spread the disease. Chemical control is a common strategy that may be applied in high-risk areas where most commercially available hybrids, or hybrids with high market value, lack a high level of resistance to this pathogen.

The current work investigates the use of a combined treatment of chemical seed coating and chemical drip line irrigation to control late wilt while applying a Real-Time PCR (qPCR)-based method for detecting and monitoring *H*. *maydis* DNA inside the host tissues [16]. The qPCR molecular tracking relies on species-specific PCR primers capable of distinguishing *H*. *maydis* from other species in the *Gaeumannomyces-Harpophora* complex based on the amplified fragment length polymorphism (AFLP) profile [34]. Seed coating is a standard method for protecting emerging maize seedlings from soil-borne fungi and has the potential of playing an essential role in defending against *H*. *maydis*, as was demonstrated earlier [16]. Since initial infection occurs during the seedling stage, this disease can be managed efficiently with seed treatment fungicides. Indeed, in a relatively resistant maize that showed only minor disease symptoms, the AS seed coating (0.0025 mg active ingredient per seed) blocked fungal progression and increased cob and plant weight [16]. However, this treatment was unable to protect a sensitive maize hybrid in heavily infested soil at the disease wilting breakout (60 DAS) and later.

In the present study, we applied the seed coating alone or in combination with the drip protection treatment. Several fungicide mixtures were injected into the drip irrigation line to test their efficiency against the disease-causing agent *H*. *maydis* in an infested maize field in the spring and summer of 2017. Earlier work done in 2009–2010 [28] in a nearby area demonstrated the effectiveness of the triple application (17, 31 and 45 DAS) of Azoxystrobin (AS, commercial name Amistar S.C.; manufactured by Syngenta, Basel, Switzerland and supplied by Adama Makhteshim, Ashdod, Israel) using a drip line deposited in each row. Although this treatment inhibited the development of wilt symptoms and recovered cob yield by 100%, it was very expensive and thus non-viable. Indeed, it was never applied on a commercial field scale in Israel.

The following work aimed at evaluating several aspects of a more cost-efficient, improved treatment, including the use of a double-row garden bed and new fungicide mixtures injected directly into the drip line in a timetable similar to before [28]. The effectiveness of this new approach on the pathogenesis of *H*. *maydis* studied in an infested field was carried out by assessing disease symptoms and wilt levels, measuring yield production and using the qPCR DNA-sequence-based approach.

## Materials and methods

### Fungal isolate and growth conditions

One representative isolate of *H*. *maydis* called *Hm-2* (CBS 133165) was selected for this study. This isolate is currently deposited in the CBS-KNAW Fungal Biodiversity Center, Utrecht, The Netherlands. Like other isolates (also deposited in CBS-KNAW in the same collection), this *H*. *maydis* strain was recovered from wilting maize plants (*Zea mays* L., Jubilee cv., Syngenta, Fulbourn, Cambridge, UK) sampled in Sde-Nehemia in the Hula Valley in the Upper Galilee (northern Israel) in 2001. The Israeli *H*. *maydis* isolates (including this representative strain) were previously characterized by their pathogenicity, physiology, colony morphology and microscopic traits [4, 35]. The microscopic and morphological characteristics of this isolate were identical to those of previously described strains found in Egypt and India [6, 36]. Final confirmation was achieved by PCR-based DNA analysis [4, 10].

All colonies were grown on potato dextrose agar (PDA) (Difco, Detroit, MI, USA) at 28±1°C in complete darkness for 4–7 days. The *H*. *maydis* pathogenic behavior that was tested in a series of experiments aimed at evaluating chemical treatments, starting from *in vitro* agar plates assay, followed by potted plants over a full growth period in a greenhouse, and eventually inspected in a field assay.

### Laboratory experiment

#### Fungicide efficiency inspection in agar plates assay

*In vitro* evaluation of eight technical-grade, commercial fungicides alone or in mixtures (Table 1) was carried out on the radial mycelial growth of *H*. *maydis*. Fungicide sensitivity was evaluated at three rates (1, 10 and 100 mg/L active ingredient) as previously described [10]. Since this is the first time that most of these fungicides were tested against the Israeli *H*. *maydis* isolate, a manufacturer’s recommendation rate was not available. Each fungicide stock solution was prepared by dissolving it in double-distilled water (DDW) according to the manufacturer’s instructions. The final stock concentration was 1000 mg/l. Media for the inhibition-response experiments were prepared by adding the fungicides in the desired concentration to an autoclaved PDA after it had chilled down to 55°C.

**Table 1:** Fungicides used in this study^a^.

Fungicide Commercial Name	Manufacturer, Supplier	Active Ingredient (common name)	Group Name	Chemical Group	Target Site of Action	Active Ingredient (g/l)	Inspected in Culture/ Plot/ Field
**Amistar** ^**b**^	Syngenta (Basel, Switzerland), Adama Makhteshim (Airport City, Israel)	Azoxystrobin (CAS no. 131860-33-8)	QoI-fungicides (quinone outside inhibitors)	Methoxy-acrylates	**Respiration** C3: cytochrome bc1 (ubiquinol oxidase) at Qo site (*cyt b gene*)	250	Culture (1–10 ppm) Pots (0.002 cm^3^/seed)
**Dividend**	Syngenta (Basel, Switzerland), Gadot Agro (Kidron, Israel)	Difenoconazole (CAS no. 119446-68-3)	DMI-fungicides (DeMethylation Inhibitors, SBI: Class I)	Triazoles	**Sterol Biosynthesis in membranes** G1: C14- demethylase in sterol biosynthesis (erg11/cyp51)	30	Culture (1–10 ppm) Pots (0.002 cm^3^/seed)
**Signum** ^**b**^**W.G.**	BASF (Ludwigshafen, Germany), Adama Agan (Ashdod, Israel)	26.7% Boscalid (CAS no. 188425-85-6) + 6.7% Pyraclostrobin (CAS No. 175013-18-0)	SDHI (Succinate dehydrogenase inhibitors) QoI-fungicides (Quinone outside Inhibitors)	Pyridine- carboxamides Methoxy-carbamates	**Respiration** C2: succinate-dehydrogenase **Respiration** C3: cytochrome bc1 (ubiquinol oxidase) at Qo site (*cyt b gene*)	267 67	Culture (1–10 ppm) Pots (0.002 cm^3^/seed)
**Ortiva top**	Syngenta (Basel, Switzerland) Adama Makhteshim (Airport City, Israel)	Azoxystrobin (CAS no. 131860-33-8)	QoI-fungicides (quinone outside inhibitors)	Methoxy-acrylates	**Respiration** C3: cytochrome bc1 (ubiquinol oxidase) at Qo site (*cyt b gene*)	250	Culture (1–10 ppm) Pots (0.002 or 0.006 cm^3^/seed) Field (0.006 cm^3^/seed and 2.25 L/hectare)
		Difenoconazole (CAS no. 119446-68-3)	DMI-fungicides (DeMethylation Inhibitors, SBI: Class I)	Triazoles	**Sterol Biosynthesis in membranes** G1: C14- demethylase in sterol biosynthesis (erg11/cyp51)	125	
**Ohayo**	Phyteurop (France) Luxembourg (Israel)	Fluazinam (CAS no. 79622-59-6)	QiI-Quinone inside inhibitors	2,6-dinitro-anilines	**Respiration** C5: Uncouplers of oxidative phosphorylation	500	Culture (1–10 ppm) Pots (0.001 cm3/seed) Field (2.25 L/hectare)
**Sportec** ^**b**^	Bayer CropScience (Monheim am Rhein, Germany) Gadot Agro (Kidron, Israel)	Prochloraz (CAS no. 67747-09-5)	DMI-fungicides (DeMethylation Inhibitors, SBI: Class I	Imidazoles	**Sterol Biosynthesis in membranes** G1: C14-demethylation in sterol biosynthesis (*erg11/cyp51*)	450	Culture (1–10 ppm) Pots (0.002 cm^3^/seed)
**Velum** **+Flint**	Bayer CropScience (Monheim am Rhein, Germany) Lidorr Chemicals Ltd. (Ramat Hasharon, Israel)	Fluopyram (Velum) (CAS no. 658066-35-4)	SDHI (Succinate dehydrogenase inhibitors)	Pyridinyl-ethyl-benzamides	**Respiration** C2: complex II: succinate-dehydrogenase	200	Culture (1–10 ppm) Field (2.25 L/hectare)
		Trifloxystrobin (Flint) (CAS no. 141517-21-7)	QoI-fungicides (Quinone outside Inhibitors)	Oximino acetates	**Respiration** C3: complex III: cytochrome bc1 (ubiquinol oxidase) at Qo site (*cyt b gene*)	500	
**Proline** **+Folicur**	Bayer CropScience (Monheim am Rhein, Germany) Lidorr Chemicals Ltd. (Ramat Hasharon, Israel)	Prothioconazole (Proline) (CAS no. 178928-70-6)	DMI-fungicides (DeMethylation Inhibitors) (SBI: Class I)	Triazolinthiones	**Sterol Biosynthesis in membranes** G1: C14-demethylation in sterol biosynthesis (*erg11/cyp51*)	275	Culture (1–10 ppm) Field (2.25 L/hectare)
		Tebuconazole (Folicur) (CAS no. 107534-96-3)	DMI-fungicides (DeMethylation Inhibitors) (SBI: Class I)	Triazoles	**Sterol Biosynthesis in membranes** G1: C14-demethylation in sterol biosynthesis (*erg11/cyp51*)	200	

^a^ This information is based on the fungicides data sheet published by the manufacturer

and the Fungicide Resistance Action Committee (FRAC) Code List 2018.

^b^ Inspected in the field in the past [28].

Fifteen milliliters of these PDA amended with different dosages of fungicides were poured into a 9 cm diameter Petri dish. Each media plate, including the control (without fungicide), was inoculated (after solidification) with a 6 mm (in diameter) colony agar disk cut from the margins of 4-6-day-old *H*. *maydis* cultures. The labeled Petri dishes were incubated at 28±1°C in the dark. All treatments (fungicide at different dosages and the control) were replicated three to six times. Radial mycelial growth was taken six days post inoculation by determining the diameter along two perpendicular lines from the underside of the Petri dishes. After subtracting the original mycelial disk diameter (6 mm) from each measurement, colony growth was converted into inhibition of growth (IG) percentage. The IG value was achieved by comparing colony size in the presence of the fungicide with colony size on the control plates using the following formula: 100 × (mean colony diameter on media amended with fungicide)/(mean colony diameter on media without fungicide). The experiment was arranged in randomized complete block design and repeated twice.

### Greenhouse experiment

#### Greenhouse experimental procedures

The effectiveness of fungicide seed coating against *H*. *maydis* at late growth stages was studied in two consecutive experiments conducted in a greenhouse in northern Israel in the Upper Galilee regional experimental farm called ‘The Plantation Farm,’ located near Kibbutz Lehavot Habashan, during the winter and spring (January-April) and summer and autumn (August-November) of 2017. The experiments were conducted with permissions of the experimental farm management for these locations and activities. The first trial included the assessment of seed coating with four selected commercial fungicides (composite with one or two active ingredients). The second experiment aimed at examining more closely the most successful treatment from the first trial (the Azoxystrobin and Difenoconazole mixture seed coating) in order to evaluate the contribution of each ingredient separately and the mixture dosage. To ensure a consistent and high level of infection, we used naturally infested soil taken from the maize field described below (mixed with 30% Perlite No. 4) to which complementary inoculation with the *Hm-2* isolate was carried out in two steps. First, 20 g of sterilized infected wheat seeds were added to the top 20 cm of the soil of each pot with the sowing. These seeds were previously incubated for 15 days at 28°C in the dark with *H*. *maydis* colony agar disks (10 disks per 100 g seeds) and were used here to spread the pathogen in the soil, as previously described [4, 33]. Second, with the above-ground appearance (on day 6 of growth), two agar disks (6-mm-diameter) from five-day-old *H*. *maydis* colonies (grown on PDA at 28°C in the dark as described above) were added to the upper parts of the roots (4 cm beneath the ground surface). Controls included unprotected plants (plants without seed coating) in contaminated soil and the same unprotected seeds in infected soil but with the complementary inoculation with *H*. *maydis* isolate as described above.

The sweet maize Prelude cv. (from SRS snowy river seeds, Australia, supplied by Green 2000 Ltd., Israel) was selected for this experiment. The Prelude cv. had been tested previously for susceptibility to late wilt [16]. Five untreated (control) or fungicide-coated seeds were planted in each 10 L pot.

The fungicides tested in this experiment are listed in Table 1. Seed coating was performed according to a typical commercial procedure by mixing the seeds with the pesticide until full cover was obtained (the fungicide was wholly absorbed on the seeds). The amount of each fungicide preparation was 0.002 cm^3^ (or 0.001 cm^3^/seed in the Fluazinam treatment) per seed (the standard concentration used by Syngenta AG, Basel, Switzerland) unless otherwise indicated. The control groups (uncoated seed plants in the inoculated ground) were grown under the same conditions. Thinning to one plant per pot was done 25 DAS. Each treatment included 10 independent replications (pots).

#### Effect of four commercial fungicide seed coatings on late wilt disease

This experiment aimed at evaluating Sportec (Prochloraz), Ohayo (Fluazinam), Signum W.G. (Boscalid and Pyraclostrobin mixture, BC+PS) and Ortiva top (Azoxystrobin and Difenoconazole mixture, AS+DC) as potential seed coating treatments to minimize late wilt disease outbreaks. The experiment was repeated twice (with similar results) and was carried out in a completely randomized design. Data are presented only for the second experiment repetition (conducted in January-April 2017).

Maize plants cv. Prelude was grown for 109 days in a greenhouse at average air temperature of 28±3°C (average ground temperature was 21±3°C) and an 8-10-h photoperiod per day. They were fertilized once (24 DAS) with 25 g of Multicote Agri per pot and watered for 15 min with 2 L (or more if required) in each pot, every 24 hours, using a computerized irrigation line system. Average male flowering was recorded on day 59 and average female flowering was recorded on day 80.

The degree of emergence of the plants in each treatment and in the controls was determined 4 DAS. Seeding emergence was considered to take place when the tip of the coleoptile was observed above-ground. Although we operated a heater inside the greenhouse to stabilize the temperature, the experiment lasted longer than usual (commonly 70–80 days) due to more limited light conditions and temperature fluctuations in winter. Dehydration assessment was done 87, 104 and 109 DAS (28, 45 and 50 days after fertilization, DAF) by calculating the percentage of plants showing typical late wilt dehydration symptoms–the color alternation of the upper leaves to light-silver and then to light-brown, and rolling inward from the edges of the entire leaf. Harvesting was carried out 109 DAS (50 DAF). At the end of the experiment, all the replications of each treatment were uprooted, and the above-ground parts of each plant were cleaned of visible soil by rinsing thoroughly under running tap water and drying gently with paper towels. For wet weight assessment, the shoot of each plant was separated by a scalpel from the root and the fresh biomass was measured individually using analytical scales. The above-ground height (from the first node to the shoot tip) of each plant was measured separately. Dry biomass of the shoot was determined after drying at 65ºC for 62 h. Tissue samples were taken from the first above-ground internode to identify the fungal DNA inside the host tissues using qPCR.

#### Effect of seed coating with Azoxystrobin and Difenoconazole on maize late wilt disease

The AS+DC antifungal compound, which was found to be the most effective in the experiment described above, was examined more intensively in the summer and autumn of the same year (2017) in the same greenhouse. This subsequent experiment was carried out according to the same design as the trial described above. Here, we repeated the AS+DC treatment, but also examined its ingredients (AS or DC) separately and evaluated the effectiveness of a triple dosage of the AS+DC mixture (0.006 cm^3^/seed). The experiment was conducted during August-November 2017, with an average temperature of 28°C and an 8-12-h photoperiod per day. The emergence degree of the experimental plants was determined 7 DAS. Fertilization day was not recorded since at the end of the experiment, most plants did not pass the vegetative stage (VT). The dehydration degree assessment was done 69 DAS as mentioned before. Determination of the phenological stage, i.e., plant height, shoot wet weight and number of green leaves, was done 72 DAS. The phenological stages were evaluated by examining the plants of each treatment, Individually, according to the maize development stages as described by Abendroth et al. [37]: VT–tasseling, R1 –silking, R2 –blister, R3 –milk, R4 –dough. At the end of this experiment (72 DAS), the plants were at the R2 (blister) stage and didn’t reach the kernel milk stage. Thus, we were unable to determine yield productivity.

### Field experiment

The field experiment was authorized and supervised by the Ministry of Agriculture and Rural Development, Consultation Service (Shaham), Beit-Dagan, and by the Israel Northern Research and Development (Northern R&D). The owner of the land, Shemesh Field Crops Partnership (Upper Galilee, northern Israel), gave permission to conduct the study on this site. The field studies did not involve endangered or protected species.

#### Effect of AS+DC seed coating and fungicide application by drip irrigation on maize late wilt disease

The field experiment aimed at assessing AS-DC seed coating and the use of four different commercial fungicides that were implemented through a drip irrigation line in controlling *H*. *maydis* pathogenesis. The sweet maize Prelude cv. was chosen as a representative susceptible cultivar. The experiment was performed during the spring and summer of 2017 in the southern area of a maize field (Mehogi 5 maize plot) near Kibbutz Amir in the Hula Valley (Shemesh Field Crops Partnership, Upper Galilee, northern Israel), which has been known to be infested with late wilt for many years [16]. This area was a separate part of a large maize field used for grain production. The cultivations included the use of a double-row garden bed (row spacing of 50 cm) compared to the standard row spacing of 96.5 cm (S1 Fig). The plants in the double-row cultivation treatment were irrigated using a one-drip line to inspect the supply of fungicide whose movement from the drip irrigation point to the plant is dependent on fungicide interactions with the soil. Such an application is considered more cost-effective because of the lower expenses involved in laying one drip line per two rows, instead of assigning one drip line per row. In the traditional row spacing (96.5 cm), we applied one irrigation drip line for each row placed adjacent to the plant rows, as was previously done [28].

The average air temperature in the experimental field throughout the experimental period (May 24-August 7, 2017) was 27.7°C, with a minimum temperature of 14.3°C and a maximum of 40.3°C (according to the Kiryat Shmona Akademia BaKikar IQIRYATS3 weather station measurements, supported by Tel-Hai College and MIGAL–Galilee Research Institute). Average humidity was 56.7% (with a minimum of 17% and a maximum of 88%). No precipitation was measured during this period.

The experiment comprised 7 treatments (unprotected control plants and six chemical treatments). Each treatment included six independent repeats (plots). The combined area of each treatment was 186 m^2^ and the total experimental area was 20.6 hectares. Plots were arranged in the field using a randomized complete block design. The area included 84 plots, each containing two rows. Each row was 16 m long and contained seven maize plants m^-1^ (approximately 112 plants per row).

The field was irrigated with a 16 mm drip line with 50 cm drip spacing (Dripnet PC1613 F, Netafim). Drip flow rate was 1 L/H and 2 L/H in the line-per-row treatment and the double-row cultivation, respectively, thus all the treatments had the same irrigation rate and even distribution of fungicides throughout the experiment. During the overall growing season, the field was irrigated with a total of 600 mm. All six treatments were irrigated together with an automatic irrigation controller (NMC Junior, Netafim). A six-outlet manifold with a specific unit at each outlet (S2 Fig) was used as a point of injection and mixing of the fungicides with the irrigation water for a homogeneous solution in each different treatment.

Seeds were pretreated (according to a standard commercial procedure by Gadot Agro, Kidron, Israel) with 0.006 cm^3^/seed AS+DC commercial preparation (the highest recommended concentration by Syngenta AG, Basel, Switzerland). Seeding was performed on May 24, 2017, and germination took place one day later by watering the field. Plants emerged above the ground surface approximately six days after planting. In the drip protection treatments, the Prelude cv. field plants were treated separately with four different commercial fungicide preparations (Table 1): Azoxystrobin and Difenoconazole mixture (Ortiva top, AS+DC); Fluazinam (Ohayo); Fluopyram (Velum) and Trifloxystrobin (Flint) mixture (FL+TR); Prothioconazole (Proline) and Tebuconazole (Folicur) mixture (PR+TE). Each fungicide or mixture was applied at a dosage of 2.25 L/hectare (83.4 cm^3^ per treatment) three times on June 8, June 29 and July 12, 2017–15, 36 and 49 days after sowing. The control groups were untreated plants and plants protected only by the AS seed coating. Flowering occurred on June 10, 2017 (47 DAS) and pollination was performed when the plants reached 70% silk on June 20, 2017 (57 DAS). First wilting symptoms in the control plants were revealed approximately one week after flowering (55 DAS), and wilt determination was carried out for all the experimental plants 70 DAS, 13 DAF (2/8/17) by calculating the percentage of plants showing typical late wilt dehydration symptoms. These symptoms included color alternation of the upper leaves to light-silver and then to light-brown, and rolling inward from the edges of the entire leaf. Yield determination included all the upper part plant cobs in a 5-m-long section of each of the experimental rows. The cobs of each plant were weighed independently, freshly. Until harvest day (August 7, 2017, 18 DAF, 75 DAS), plots in the control treatment collapsed and severe yield loss was recorded.

### Soil solution sampling and evaluation

The soil solution of the AS+DC and FL+TR treatments was sampled using a MacroRhizon soil moisture sampler (no. 19.21.35/36, Giesbeek, The Netherlands). Sampling was done using a 50 ml syringe + spacer with a mean pore size of 0.1 microns according to the manufacturer’s instructions. The sampling point was selected arbitrarily in the middle of the treatment line in three independent and relatively distant repeats. Samples were taken approximately 10 cm beneath the ground surface at a distance of 0, 14 and 28 cm from the drip irrigation point (S3 Fig), immediately after the second and third administrations of fungicides, on June 29 and July 12, 2017–36 and 49 DAS, respectively. The soil solution was collected 72 h after injection of the fungicides, with a typical yield of 5–7 ml. Fungicide concentration in the ground solutions was tested using the colony agar plates assay described above. In this bioassay, soil solutions were filtered using a 0.2 μm syringe filter, and 4 ml from the filtrate was added to a 5 ml autoclaved PDA. Colony growth rate was determined three days post inoculation under the conditions mentioned above. The plate method was used to determine fungicide concentration in the ground. Each analysis included an untreated control. The effectiveness of the fungicide (IG value) was determined, as described above.

### Molecular diagnosis of late-wilt pathogen

Six plants were collected arbitrarily from each treatment at three intervals from sowing onwards. Sampling was carried out on days 29 (22/6/17, roots), 50 (13/7/17, roots and stems) and 70 (2/8/17, stems) after seeding. Different plant tissues (roots or stems) were cleaned of visible soil by rinsing thoroughly under running tap water. Tissue samples were taken by removing a cross-section of approximately 2 cm in length from each plant; total weight was adjusted to 0.7 g and considered to be one repeat. Tissue samples were placed in universal extraction bags (Bioreba, Switzerland) with 4 ml CTAB buffer, and the tissue was ground with a hand tissue homogenizer (Bioreba, Switzerland) for 5 min until the tissues were entirely homogenous. The homogenized samples were processed for DNA purification, as described below.

### DNA extraction and qPCR

Total DNA preparations were obtained from tissue samples of axenically grown maize tissue and from maize tissue known to be infected with *H*. *maydis* using a slight modification of the procedure of [38]. After grounding the tissue with 4 ml CTAB buffer (0.7 M NaC1, 1% cetyltriammonium bromide (CTAB), 50 mM Tris-HC1 pH 8.8, 10 mM EDTA and 1% 2-mercaptoethanol), 1.2 ml from the mixture was incubated for 20 minutes at 65°C. The samples were then centrifuged at 14,000 rpm for 5 min at room temperature (24°C). The upper phase of the lysate (usually 700 μl) was then extracted with an equal volume of chloroform/isoamyl-alcohol (24:1). After mixing by vortex, the blend was centrifuged again at 14,000 rpm for 5 min at room temperature. This stage of chloroform/isoamyl-alcohol extraction was repeated twice. The supernatant (usually 300 μl) was then separated to a new Eppendorf tube and mixed with cold isopropanol (2:3). The DNA solution was mixed gently by inverting the tube several times, kept at -20°C for 20–60 min, and centrifuged (14,000 rpm in 4°C for 20 min). The precipitate DNA was isolated and resuspended in 0.5 ml 70% ethanol. After another centrifugation (14,000 rpm at 4°C for 10 min), the precipitate DNA was isolated and left to dry in a sterile hood overnight. Finally, the DNA was suspended in 100 μl HPLC-grade water and kept at 20°C until use.

*qPCR-based method*. All qPCR reactions were performed, as previously described [16], using the ABI PRISM 7900 HT Sequence Detection System (Applied Biosystems, California, USA) for 384-well plates. Final qPCR conditions were as follows: 5 μl total reaction volume was used per sample well– 2 μl of sample DNA extract, 2.5 μl iTaq Universal SYBR Green Supermix (Bio-Rad Laboratories Ltd., Rishon Le Zion, Israel), 0.25 μl forward primer and 0.25 μl reverse primer (10 μM from each primer to a well). The final PCR parameters were as follows: precycle activation stage, 1 min at 95°C; 40 cycles of denaturation (15 s at 95°C), and annealing and extension (30 s at 60°C), followed by melting curve analysis. Plant tissue samples (root and stem) from each experiment were analyzed separately by qPCR. The A200a primers were used for quantitative Real-Time PCR (qPCR) (sequences in Table 2). The gene coding for the last enzyme in the respiratory electron transport chain of the eukaryotic mitochondria–cytochrome c oxidase (*COX*)–was used as a “housekeeping” reference gene to normalize the amount of DNA [39]. This gene was amplified using the COX F/R primer set (Table 2). Relative gene expression was calculated according to the ΔΔCt model [40]. Efficiency was assumed to be the same for all samples. All amplifications were performed in triplicate.

**Table 2:** Primers used in this study for *Harpophora maydis* detection.

Pairs	**Primer**	**Sequence**	**Uses**	**Amplification**	**References**
**1**	A200a-for A200a-rev	5’-CCGACGCCTAAAATACAGGA-3’ 5’-GGGCTTTTTAGGGCCTTTTT-3’	qPCR	*H*. *maydis* AFLP-derived species-specific fragment	[4]
**2**	Cox-F Cox-R	5’-GTATGCCACGTCGCATTCCAGA-3’ 5’-CAACTACGGATATATAAGRRCCRRAACTG -3’	qPCR control	cytochrome c oxidase (COX) gene	[39]

### Statistical analyses

When assessing the *H*. *maydis* in agar plates assay (IG values) or the infection outcome on symptoms in the greenhouse in mature plants’ infection or in diseased field plants, we used fully randomized statistical designs. Student’s t-test (with a significance threshold of *P* = 0.05) was used for comparisons of treatment means to the control.

## Results

### Laboratory experiment

#### Plate sensitivity assay for evaluating potential fungicides *in vitro*

The antifungal activity of AS was tested in a mixture with DC against other fungicide commercial mixtures in a plate sensitivity assay (Table 3). The growth inhibition of the fungus increased depending on the concentration of the active ingredient. The control plates reached about 15 mm of growth per day. All the agar-embedded, anti-fungal compounds tested in this assay led, at a 100 ppm dosage, to high and significant (*P* < 1.0E-03) suppression of *H*. *maydis* growth. The fungicide mixtures were especially effective (93% IG, *P* = 3.8E-05, at 100 ppm).

**Table 3:** Fungicide efficiency agar plate assay.^a^.

Fungicide Commercial Name	Active Ingredient	Dose (ppm)	Inhibition of Growth (IG) (% ± S.E.)	Statistical Difference from the Control
**Velum**	Fluopyram (FL)	1	46.7 ± 0.00	1.0E-03
		10	46.7 ± 0.00	1.0E-03
		100	49.0 ± 0.55	8.1E-04
**Amistar**	Azoxystrobin (AS)	1	57.4 ± 0.72	3.7E-04
		10	58.4 ± 0.21	3.7E-04
		100	58.2 ± 2.09	2.2E-04
**Ohayo**	Fluazinam	1	81.8 ± 0.00	7.4E-05
		10	87.2 ± 0.21	5.3E-05
		100	89.8 ± 0.21	4.6E-05
**Velum + Flint**	FL + Trifloxystrobin (TR)	1	78.0 ± 0.00	9.2E-05
		10	78.9 ± 0.55	8.2E-05
		100	93.1 ± 0.36	3.8E-05
**Dividend**	Difenoconazole (DC)	1	83.0 ± 0.74	1.1E-09
		10	91.3 ± 0.45	5.3E-11
		100	94.6 ± 0.34	1.2E-11
**Ortiva top**	AS + DC	1	86.1 ± 0.35	3.0E-05
		10	94.8 ± 0.00	2.8E-05
		100	94.8 ± 0.00	2.8E-05
**Signum W.G.**	Boscalid + Pyraclostrobin (BC+PS)	1	82.9 ± 0.35	3.0E-05
		10	94.8 ± 0.00	2.8E-05
		100	94.8 ± 0.00	2.8E-05
**Proline**	Prothioconazole (PR)	1	74.5 ± 0.42	1.1E-04
		10	92.9 ± 0.42	3.8E-05
		100	96.0 ± 0.21	3.3E-05
**Sportec**	Prochloraz	1	96.2 ± 0.00	3.3E-05
		10	96.9 ± 0.00	3.2E-05
		100	96.9 ± 0.00	3.2E-05
**Proline + Folicur**	PR + Tebuconazole (TE)	1	92.3 ± 0.42	3.9E-05
		10	96.9 ± 0.00	3.2E-05
		100	96.9 ± 0.00	3.2E-05

^a^ Inhibition of the colony growth of *Harpophora maydis* was made upon amending potato dextrose agar culture media with different doses of fungicide active ingredient. Inhibition of *H*. *maydis* mycelial growth was evaluated six days after incubation by measuring the increase in colony diameter. Colony growth was converted into inhibition of growth (IG) percentage–comparing colony size in the presence of the fungicide with colony size on the control plates using the following formula: 100 × (mean colony diameter on media amended with fungicide)/(mean colony diameter on media without fungicide). Results are a mean of six replicate assays. Variations are standard errors.

One of the most effective treatments was the AS+DC, which blocked fungal growth almost completely (95% IG, *P* < 2.8E-05, at 100 ppm). This mixture treatment was noticeably more efficient than AS alone (58% IG, *P* = 2.2E-04) but nearly identical to DC alone (95% IG, *P* = 1.2E-11) at the same dose. Other fungicides such as Prothioconazole (PR) alone or combined with Tebuconazole (TE), Prochloraz or the BC+PS mixture proved to be powerful inhibitors as well (IG > 94.8, *P* ≤ 3.3E-05, at 100 ppm, Table 3). Four of them were evaluated in a subsequent greenhouse plant assay, as detailed below.

### Greenhouse experiment

#### Effect of fungicide seed coating on mature maize plants

Naturally infested soil taken from a maize field was chosen to estimate the influence of seed coating in a greenhouse over a full plant growth period. While the infection with *H*. *maydis* or the seed coating with three of the four fungicides tested did not affect sprout emergence, the AS+DC preparation caused prominent (43%) and significant (*P* = 0.04) delay in emergence compared to the non-treated infected control (Table 4).

**Table 4:** Effect of fungicide seed coating on plant development in greenhouse upon inoculation with *Harpophora maydis*^a^.

Treatment	Emergence (%)	Phenological Stage	Plant Height (cm)	Shoot Wet Weight (g)	Cob Wet Weight (g)
**Control**	40.0 ± 10.33	VT– 00% R1–30% R2–30% R3–10% R4–30%	144.0 ± 4.1	92.2 ± 6.6	46.2 ± 12.8
**Control + *H*. *maydis***	56.0 ± 8.84	VT– 20% R1–30% R2–10% R3–10% R4–30%	128.3 ± 9.4	69.2 ± 10.6	35.0 ± 10.3
**Sportec (Prochloraz)**	42.0 ± 10.09	VT– 10% R1–20% R2–10% R3–00% R4–60%	143.0 ± 4.5	79.1 ± 10.7	48.3 ± 12.4
**Ohayo (Fluazinam)**	46. 0 ± 9.91	VT– 00% R1–20% R2–20% R3–00% R4–60%	138.1 ± 6.6	76.3 ± 12.0	53.7 ± 9.0
**Signum W.G.** **(BC+PS)**	44.0 ± 10.67	VT– 10% R1–10% R2–00% R3–10% R4–70%	142.8 ± 4.4	64.1 ± 11.6	57.2 ± 11.4
**Ortiva top (AS+DC)**	32. 0 ± 6.11 (*P* = 0.04)	VT– 00% R1–00% R2–20% R3–20% R4–60%	145.1 ± 2.5	61.7 ± 7.8	65.6 ± 7.4 (*P* = 0.01)

^**a**^ Emergence percentage was evaluated 4 days after sowing (DAS). All other phenotypes were evaluated at the end of the experiment 109 DAS. The control includes unprotected plants grown on naturally infested soil. The control + *H*. *maydis* treatment was done with the addition of complementary inoculation. All other treatments were carried out in the infested soil with complementary inoculation. Results are a mean of 10 independent replicates. Variations are standard errors. Numbers in brackets represent significance difference from the control + *H*. *maydis* treatment.

The infection with *H*. *maydis* negatively affected the above-ground plant parts’ wet weight. Whereas some fungicides (Prochloraz and Fluazinam) caused insignificant improvement in this measure, the AS+DC and BC+PS treatments caused a greater reduction in shoot wet weight (found significant only compared to the control—unprotected plants grown on naturally infested soil, at *P* = 0.01 and 0.05, respectively). However, this effect was not reflected in plant height and cob wet weight measurements at the end of the growth period. On the contrary, all of the protective treatments achieved plant heights similar to the healthy plant state. Moreover, the chemical treatments achieved improved growth– 60–70% of the plants reached the R4-dough growth stage, while most of the control plants remained in the former stages.

This positive tendency was also reflected in yield production. The BC+PS treatment resulted in a 63% increase in average cob weight compared to the non-treated infected control (although this difference was not statistically significant, *P* = 0.16). In particular, the AS+DC seed coatings achieved improved results (87% average cob weight increase, Table 4), which were also statistically significant (*P* = 0.01).

Tracking *H*. *maydis* DNA inside the experiment’s host plants reveal that the Prochloraz and Fluazinam seed coating resulted in similar results to the non-treated infected control plants. However, the coatings of the AS+DC and BC+PS mixtures resulted in a lower pathogen DNA presence in the plant (Table 5). The qPCR evaluation revealed 33% and 22% less identification, respectively, of *H*. *maydis* DNA in the plant samples compared to the control. No statistical significance could be identified between the treatments and the control since the infection was not uniform, and there were some plants that were not (or only slightly) infected in every treatment. This difference was also reflected in the assessment of wilting symptoms (Table 5).

**Table 5:** The efficiency of fungicide seed coatings on late wilt in a greenhouse^a^.

	qPCR (%)	Phenotype (%)		
Treatment	Identification of *H*. *maydis* DNA 109 DAS	Wilting 87 DAS	Wilting 104 DAS	Wilting 109 DAS
**Control**	90	0.0 ± 0.0	22.5 ± 14.8	40.0 ± 23.4
**Control + *H*. *maydis***	80	15.0 ± 11.8	32.5 ± 20.7	42.5 ± 25.1
**Sportec (Prochloraz)**	90	20.0 ± 13.8	30.0 ± 19.8	45.0 ± 26.1
**Ohayo (Fluazinam)**	80	15.0 ± 11.8	25.0 ± 17.2	35.0 ± 18.3
**Signum W.G. (BC+PS)**	70	10.0 ± 7.4	40.0 ± 22.6	57.5 ± 31.7
**Ortiva top (AS+DC)**	60	0.0 ± 0.0	27.5 ± 14.9	52.5 ± 22.8

^a^ Control–plants without seed coating in contaminated field soil. The control + *H*. *maydis*–the same unprotected seeds in infested soil, but with complementary inoculation. All other treatments were carried out in the infested soil with complementary inoculation. Results are a mean of 10 independent replicates. Variations are standard errors.

At the age of 87 days, the BC+PS and AS+DC seed coatings were the only efficient treatments, and they reduced wilt symptoms by 30% and 100%, respectively (compared to the infected control). At day 104 onwards with an increase in the severity of symptoms, the differences from the control started to blur. A positive effect of the chemical coating was achieved only in the Fluazinam and AS+DC treatments (17% and 8% decrease, respectively). On day 109 from sowing when the wilting symptoms peaked, all of the treatments failed to protect the plants, except for the Fluazinam treatment, which had a minor, statistically insignificant influence (13% reduction in dehydration). Indeed, the dehydration symptoms increased significantly between 87 DAS and 104 DAS in the control (*P* = 0.04), BC+PS (*P* = 0.03) and AS+DC (*P* = 0.02) treatments. This difference increased dramatically in these treatments after another 5 days (*P* = 0.01, 0.004 and 0.0005, respectively).

Interestingly, the most effective treatment–the AS+DC mixture–had the most dramatic increase in wilt symptoms over time. This elevation in wilt symptoms also expresses an increase in statistical difference from 87 DAS, as described above. Nevertheless, the number of completely dry plants reached 10% in the AS+DC seed-coated plants, while 30% of the unprotected control plants were completely dry.

In a continuation experiment, we inspected the contribution of the AS+DC mixture ingredients separately and the influence of this mixture at high dosages (Table 6). The AS+DC mixture seed coating at a standard dosage did not result in improved resistance to late wilt compared to the untreated control in any of the measures examined.

**Table 6:** Effect of azoxystrobin and difenoconazole seed coating on plant development in a greenhouse upon inoculation with *Harpophora maydis*^a^.

Treatment	Emergence (%)	Phenological Stage ^a^	Plant Height (cm)	Shoot Wet Weight (g)	No. of Green Leaves
**Control + *H*. *maydis*** ^**b**^	88.0 ± 6.8	VT– 60% R1–10% R2–30%	117.3 ± 6.8	60.0 ± 9.5	2.0 ± 0.9
**Difenoconazole**	57.8 ± 9.1 (*P* = 0.02)	VT– 22% R1–22% R2–56%	137.7 ± 7.8	89.8 ± 9.9 (*P* = 0.04)	3.2 ± 1.0
**Azoxystrobin**	60.0 ± 7.5 (*P* = 0.01)	VT– 44.5% R1–44.5% R2–11%	118.7 ± 10.1	75.5 ± 7.8	4.0 ± 1.1
**AS+DC**	82.0 ± 5.5	VT– 80% R1–20% R2–00%	114.3 ± 3.5	60.8 ± 10.7	1.8 ± 0.9
**AS+DC x3**	68.0 ± 8.5	VT– 80% R1–00% R2–20%	100.9 ± 8.9	54.9 ± 7.6	3.3 ± 1.1

^a^ All fungicides used for seed coating were applied at a dosage of 0.002 cm^3^/seed, except for the triple dosage of the AS+DC mixture (AS+DC x3), which was done at a dosage of 0.006 cm^3^/seed. Emergence percentage was evaluated 7 DAS. All other phenotypes were evaluated at the end of the experiment, 72 DAS. The control + *H*. *maydis* treatment was carried out with unprotected seeds planted in naturally infested soil and the addition of complementary inoculation. Results were calculated from a mean of 10 independent replicates. Variations are expressed as standard errors.

The coating with each ingredient, AS or DC alone, significantly reduced the emergence of the plants (*P* = 0.01 and 0.02, respectively). Yet, the plant height, shoot biomass and number of green leaves measured at the end of this experiment (69 DAS) all improved remarkably following these treatments. The AS or DC treatments caused a 26% and 50% increase, respectively, in shoot weight (*P* = 0.04 in the DC treatment), and a 100% and 60% increase, respectively, in the number of green leaves. The plant development following AS or DC treatment also increased, and most of them (55.5% and 78%, respectively) passed the VT tasseling stage (60% of the plants in the control remained at this stage). In the DC treatment, 56% of the plants reached the R2 blister stage.

**Fig 1:**
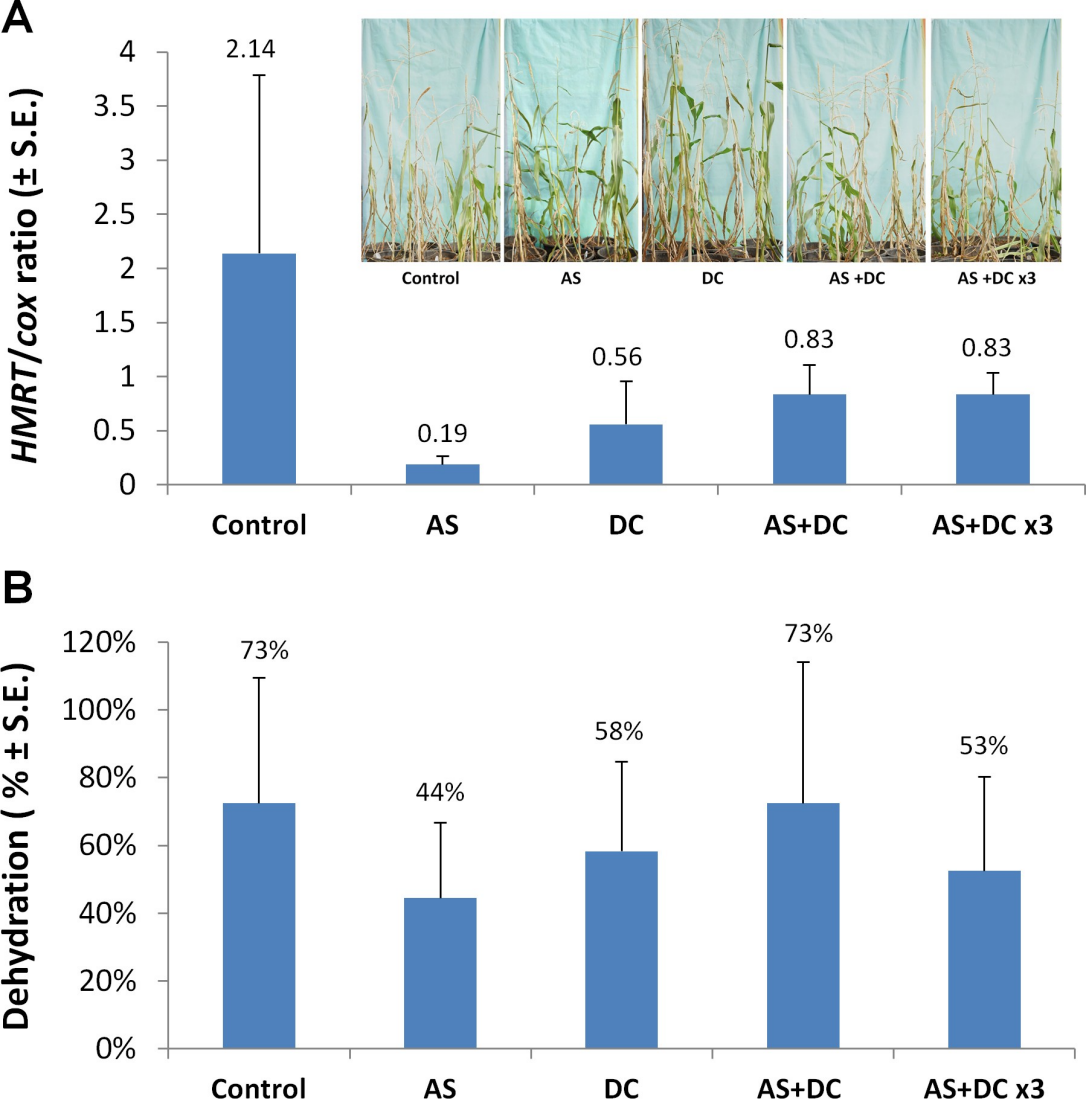
Effect of Azoxystrobin and difenoconazole seed coatings on plant development in a greenhouse. A. Real-Time qPCR analysis of *H*. *maydis* unique oligonucleotide conducted 72 DAS. The y-axis indicates *H*. *maydis* relative DNA (HMRT) abundance normalized to the cytochrome c oxidase (*COX*) DNA. B. dehydration assessment carried out 69 DAS by calculating the percentage of plants showing typical late wilt dehydration symptoms–the color alternation of the upper leaves to light-silver and then to light-brown, and rolling inward from the edges of the entire leaf. Insert–a photograph of the above-ground parts of all of the plants in each treatment, 72 DAS. Values indicate an average of 10 replicates. Error bars indicate standard error.

Moreover, the AS or DC seed coatings led to very low *H*. *maydis* DNA quantities (91% and 74% fungal DNA inhibition, respectively) (Fig 1A). In this experiment, the disease symptoms were more severe, with 73% dehydration at 69 DAS in the control (compared to only 42% dehydration in the control at 109 DAS in the first experiment). Here, again, the AS+DC mixture failed to protect the plants at the end of the experiment (and even worsened the symptoms outcome) (Table 6, Fig 1B). However, the AS+DC treatment positively affected the spreading of fungal DNA and managed to decrease 61% of it at the end of the experiment. A tripled dosage of AS+DC mixture yielded better results compared to the regular AS+DC dosage, reflected mainly in 65% more green leaves and 27% lower dehydration. This treatment caused a decrease in fungal DNA at the regular AS+DC dosage.

### Field experiment

#### Effect of fungicide seed coatings on late wilt

The influence of a combined treatment of seed coating and drip irrigation fungicide implementation against late wilt was assessed for the first time in the field. The bioassay (growth inhibition plate assay) made of the water samples at varying distances from the irrigation point (S3 Fig) revealed that the short row spacing (50 cm instead of 96 cm) of the double-row cultivation maintained sufficient fungicide concentration in the ground (Table 7).

**Table 7:** Fungicide activity in the ground–*Harpophora maydis* inhibition bioassay.

Fungicide Commercial Name	Active Ingredient	Distance from the Irrigation Point (cm)	Inhibition of Growth (IG) 36 DAS (% ± S.E.)	Statistical Difference from the Control	Inhibition of Growth (IG) 49 DAS (% ± S.E.)	Statistical Difference from the Control
**Velum + Flint**	FL + TR	0	54.2 ± 0.54	9.8E-03	35.1 ± 0.72	0.09
		14	16.9 ± 096	0.17	35.5 ± 0.24	0.34
		28	13.6 ± 1.22	0.23	37.1 ± 0.00	0.47
**Ortiva top**	AS + DC	0	75.4 ± 0.42	3.0E-03	53.2 ± 4.13	0.33
		14	62.7 ± 0.47	5.9E-03	52.4 ± 2.25	0.39
		28	59.3 ± 1.15	0.07	27.4 ± 0.00	0.50

At the second injection of fungicides (36 DAS), the IG value of the AS+DC solution sampled near the irrigation point was 75%. At the more remote distance of 14 and 28 cm from the irrigation line, the AS+DC solution maintained 63% and 59% inhibition capability, respectively. At that growth stage (36 DAS), the FL+TR solution sampled near the irrigation line maintained 54% inhibition capability, but at the distances of 14 and 28 cm from the irrigation line, the effectiveness of the FL+TR treatment dropped to 17% and 14% inhibition, respectively (Table 7). The soil moisture collected after the third fungicide injection (49 DAS) achieved a less prominent result in the plate inhibition assay. The results at that date were most certainly affected by high temperatures and high evaporation rates on that particular day. Indeed, the average temperature on 36 DAS was 26.8°C (with a peak of 34.1°C at midday), while the average temperature on 49 DAS was 31.5°C (with a peak of 36.9°C at midday). The humidly on both days was similar, an average of 50%. Moreover, the soil in the third fungicide injection day (49 DAS) was dryer than the soil in the second injection of fungicides (36 DAS). This was reflected in less water yield–about 5 ml instead of 7 ml, collected 72 h after injection of the fungicides.

**Fig 2:**
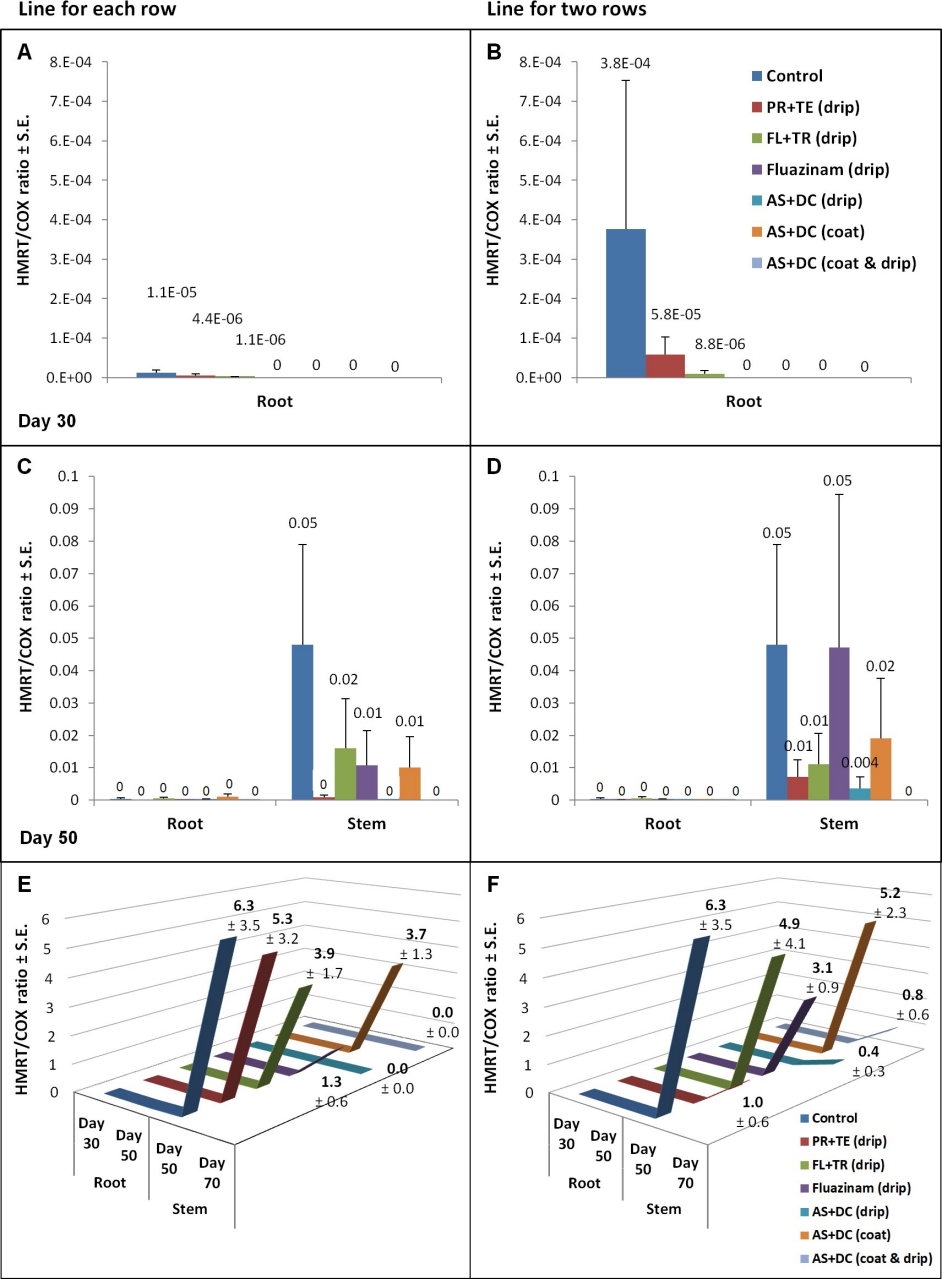
qPCR diagnosis of late wilt pathogenesis in the field (2017). Plant samples were collected arbitrarily on 30 DAS (A, B, root), 50 DAS (C, D, root and stem) and 70 DAS (E, F, stem). DNA was extracted from the plant tissues and analyzed in three independent replications. qPCR was performed to amplify a specific *H*. *maydis* segment. The y-axis indicates *H*. *maydis* relative DNA (HMRT) abundance normalized to the cytochrome c oxidase (*COX*) DNA. Bars indicate a mean of six replicates. Standard errors are indicated in error bars or in brackets in gray.

Plant samples were collected after 29 days (roots), 50 days (roots and stems) and 70 days (stems). DNA was isolated from each sample for subsequent diagnosis using the qPCR-based method. The qPCR-based molecular diagnosis enabled us to detect variations in fungal DNA inside the host roots as soon as we made the first sampling on 29 DAS. The amount of fungal DNA (reflected as *Hm/cox* ratio) in the non-protected control plants increased dramatically during the growth session, from a value of 1.1e^-5^ on 29 DAS in the root to 4.1e^-4^ on 50 DAS in the root, to 4.8e^-2^ at this age in the stalk, and eventually to a peak of 6.3 on 70 DAS in the stalk (Fig 2). These 5e^+5^ times increases were measured in the line-per-row treatment. In the double-row cultivation, fungal DNA levels were 10 times higher than the regular row spacing cultivation on the first measure (29 DAS, 3.8e^-4^), but later the difference decreased, and the DNA level elevation of the control untreated plants was similar in both cultivation methods.

The AS+DC seed coating alone managed to delay the pathogen spread in the maize tissues in the early stages of the growth season (up to the age of 50 days from sowing, Fig 2A–2D). Specifically, in the line-per-row treatment, the seed coating caused an 80% reduction in fungal DNA at this age. In comparison, the seed coating in the double-row cultivation led to a 60% DNA level reduction at this age (Fig 2C and 2D). However, the seed coating alone was unable to fully protect the crops later, and the influence on *H*. *maydis* DNA levels in this treatment dropped to a reduction of 41% and 17%, respectively, compared to the untreated control on 70 DAS (Fig 2E and 2F). All of the fungicides injected into the drip line led to lower levels of the pathogen inside the root and stem of the host maize plants. In particular, drip protection with AS+DC (alone or in combination with seed coating) was the most successful treatment, and it reduced fungal DNA in the root and shoot host tissues to near zero levels (Fig 2). This result was similar in both row-spacing growth methods, with a slightly less prominent outcome in the double-cultivation method.

**Fig 3:**
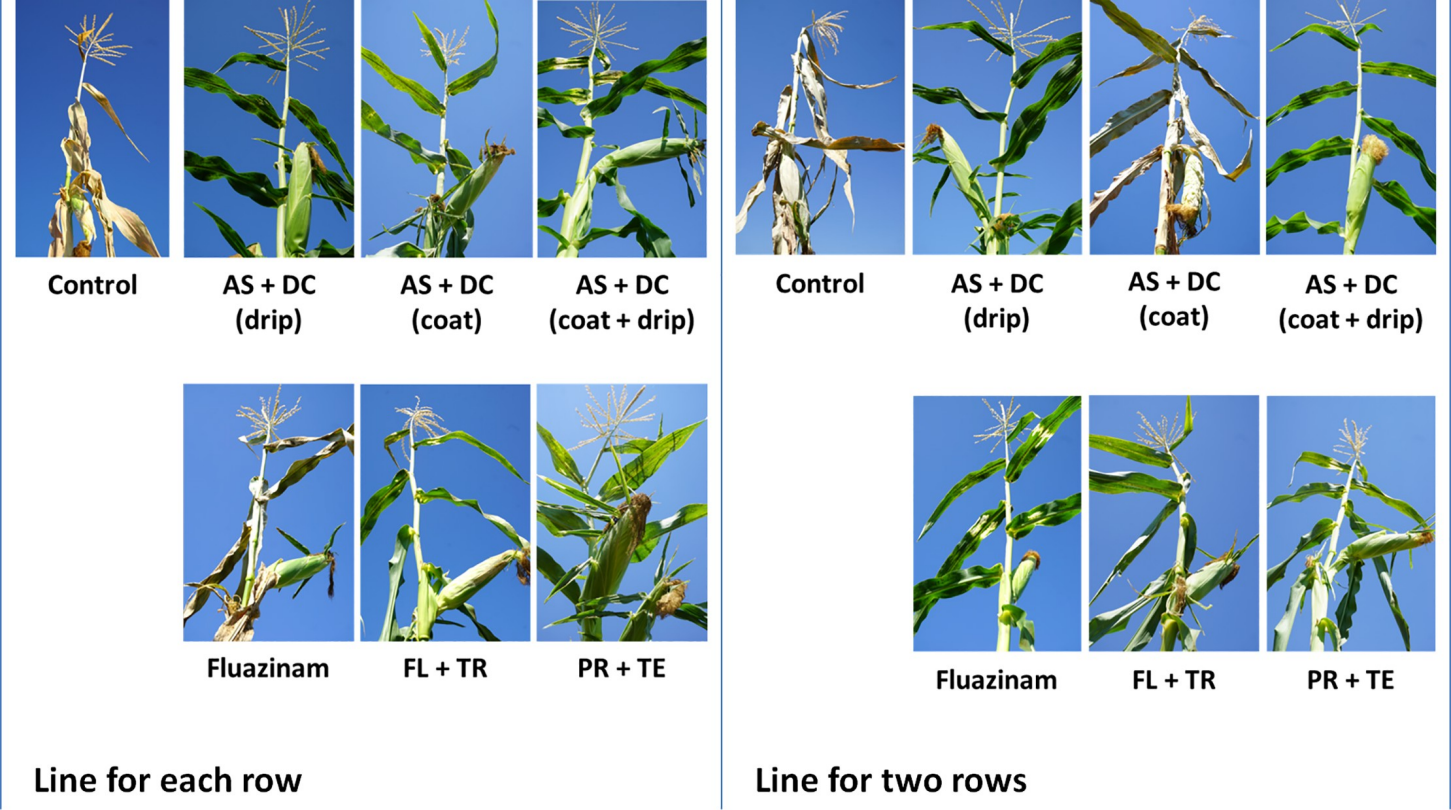
Late wilt disease symptoms in representative plants from the field experiment. Representative plants from the field experiment were collected arbitrarily and photographed 70 DAS, 13 days after fertilization (DAF). Wilt symptoms include drying out upwards in the plant, stem and leaf yellowing, and dehydration and tilt down of the cobs.

**Fig 4:**
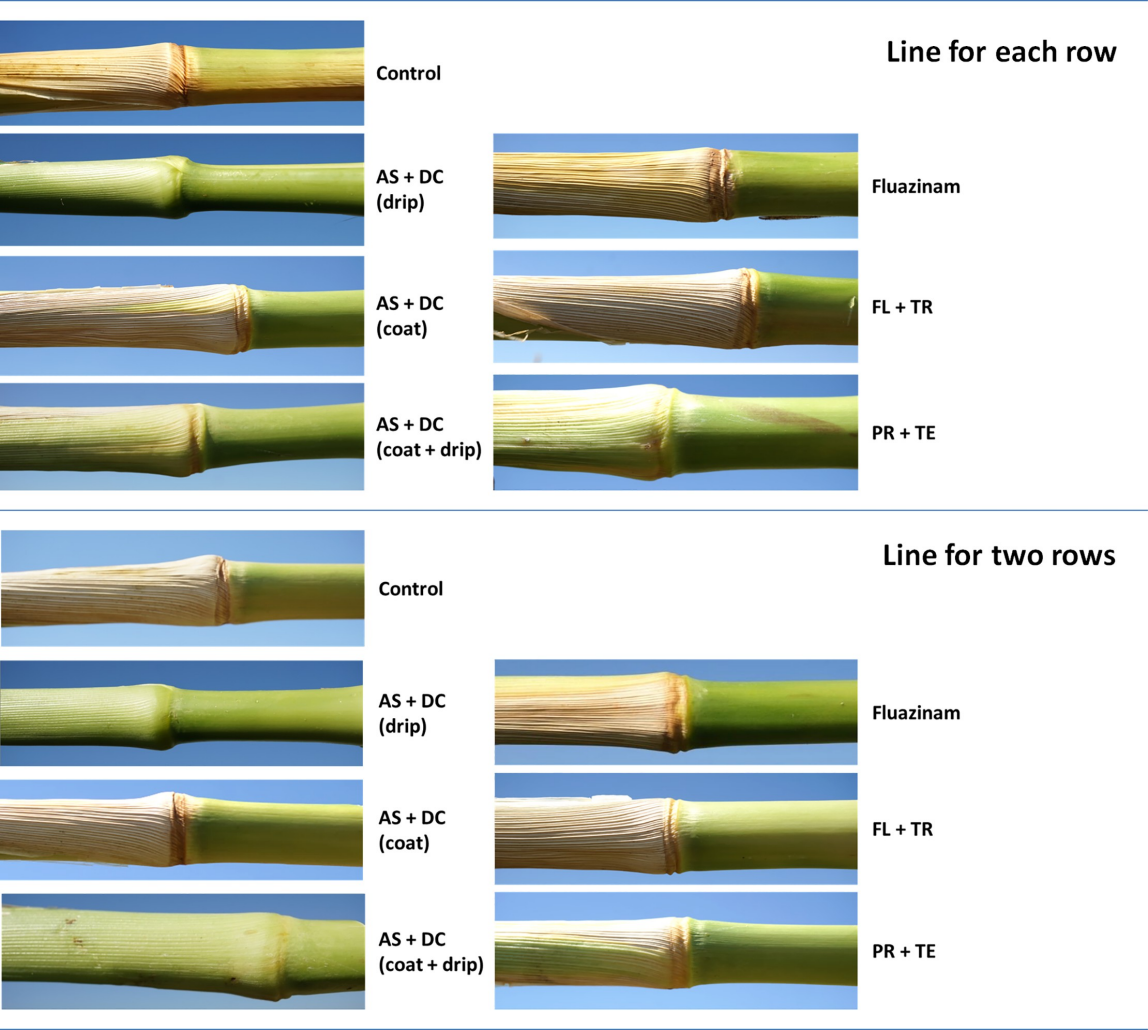
Stem surface symptoms in the field experiment. Color alteration of the Prelude cultivars’ lower stems (including the first above-ground internode). Magnification of the lower stem was made of representative plants from the field experiment at 70 DAS (13 DAF). Wilting symptoms include color alteration of the lower stems’ surface.

**Fig 5:**
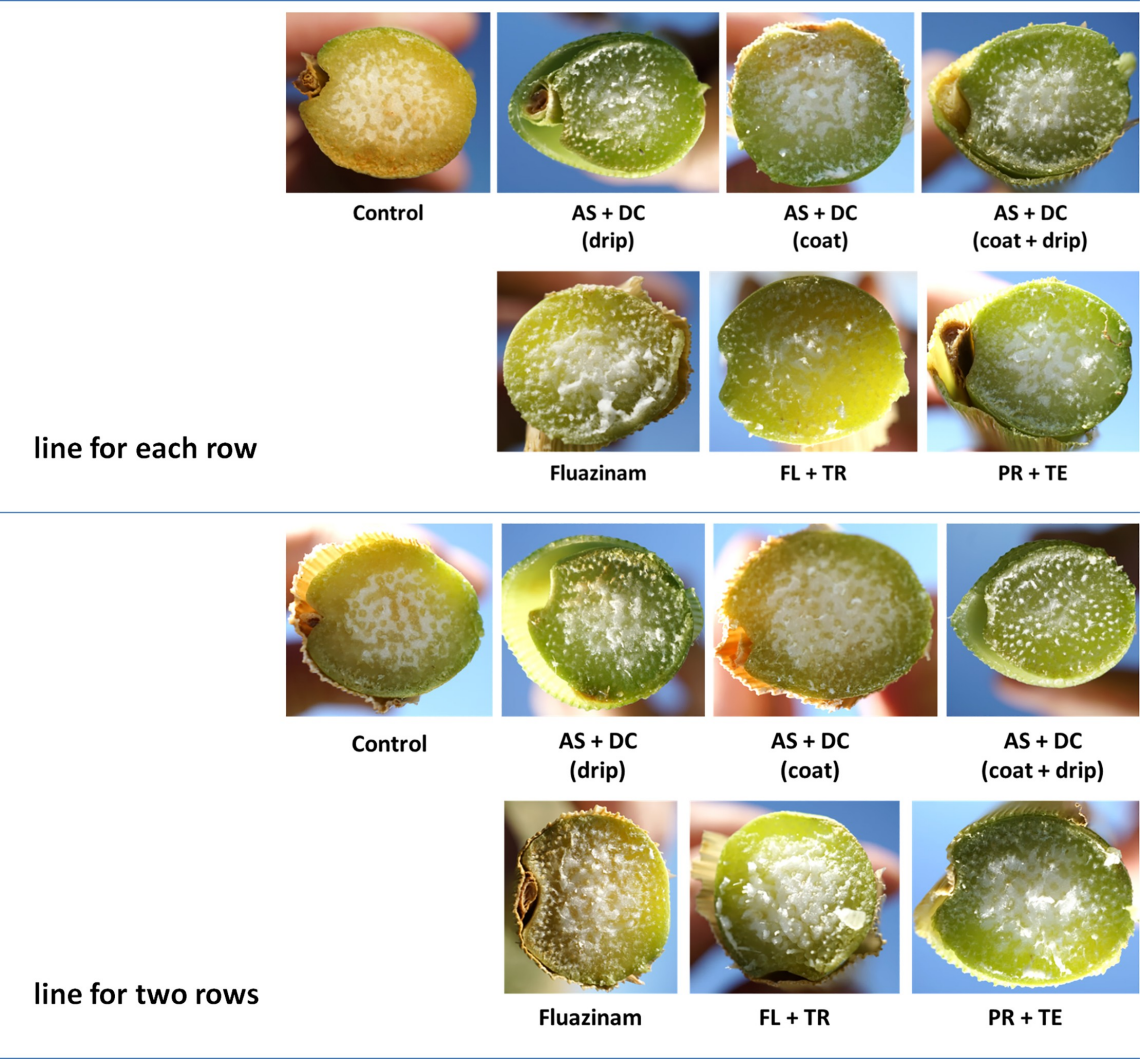
Stem cross-section symptoms in the field experiment. Cross-section magnification was made of the lower stems (below the first internode) of representative plants from the field experiment. Late-wilt symptomatic plants revealed a color alteration in the lower stems to a yellow-brown hue and vascular bundles occlusion. Photos were taken 70 DAS (13 DAF).

**Fig 6:**
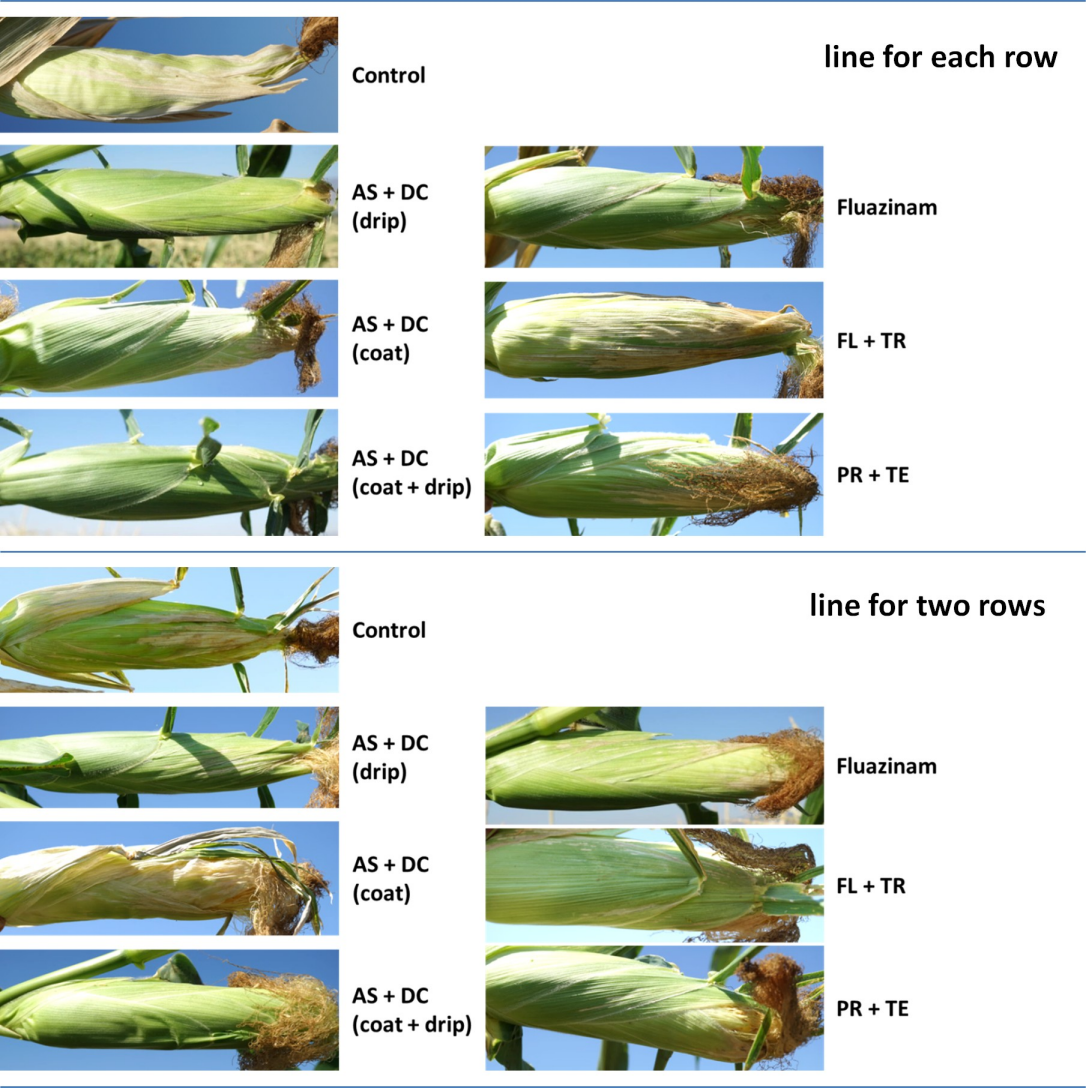
Cob symptoms in the field experiment. Photos of the cobs were taken from the plants shown in Fig 3 70 DAS and 13 DAF. Wilted plants show dehydration of the outer surface of the cobs, as well as shrinking and tilting down of the cobs.

**Fig 7:**
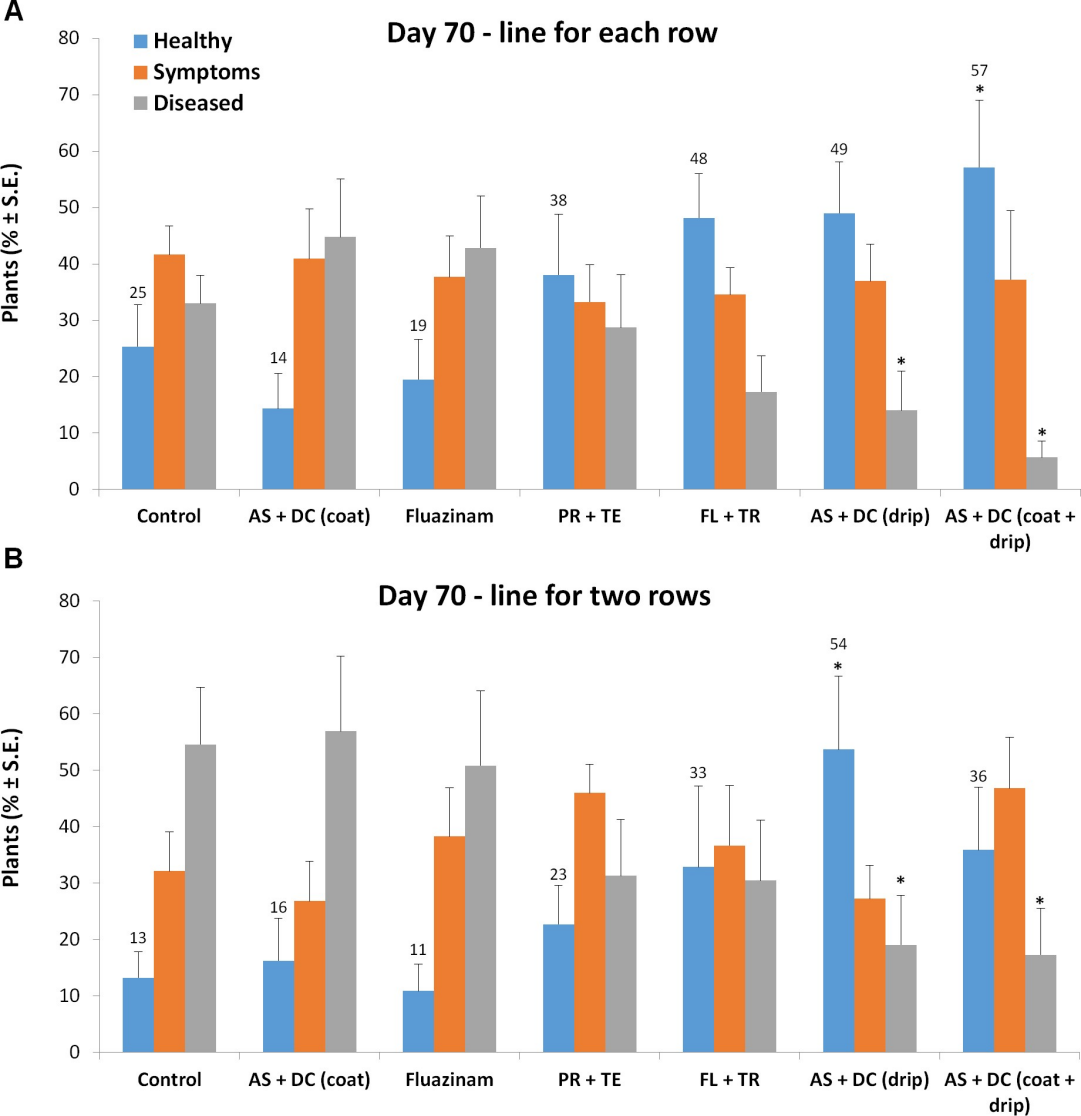
Wilt assessment of the field experiments carried out 13 days after fertilization. Wilting percentages of the field experiment were determined 70 DAS for the standard row spacing of (96.5 cm) treatment (A) and the double-row garden bed (B, row spacing of 50 cm). Plants were classified as “symptoms” when wilt symptoms appeared on the leaf whose cob was located in its lap or “diseased” when the entire cob dried out. Vertical upper bars represent the standard error of the mean of six replications. Significance from the control (untreated plants) is indicated as * = *P* < 0.05.

**Fig 8:**
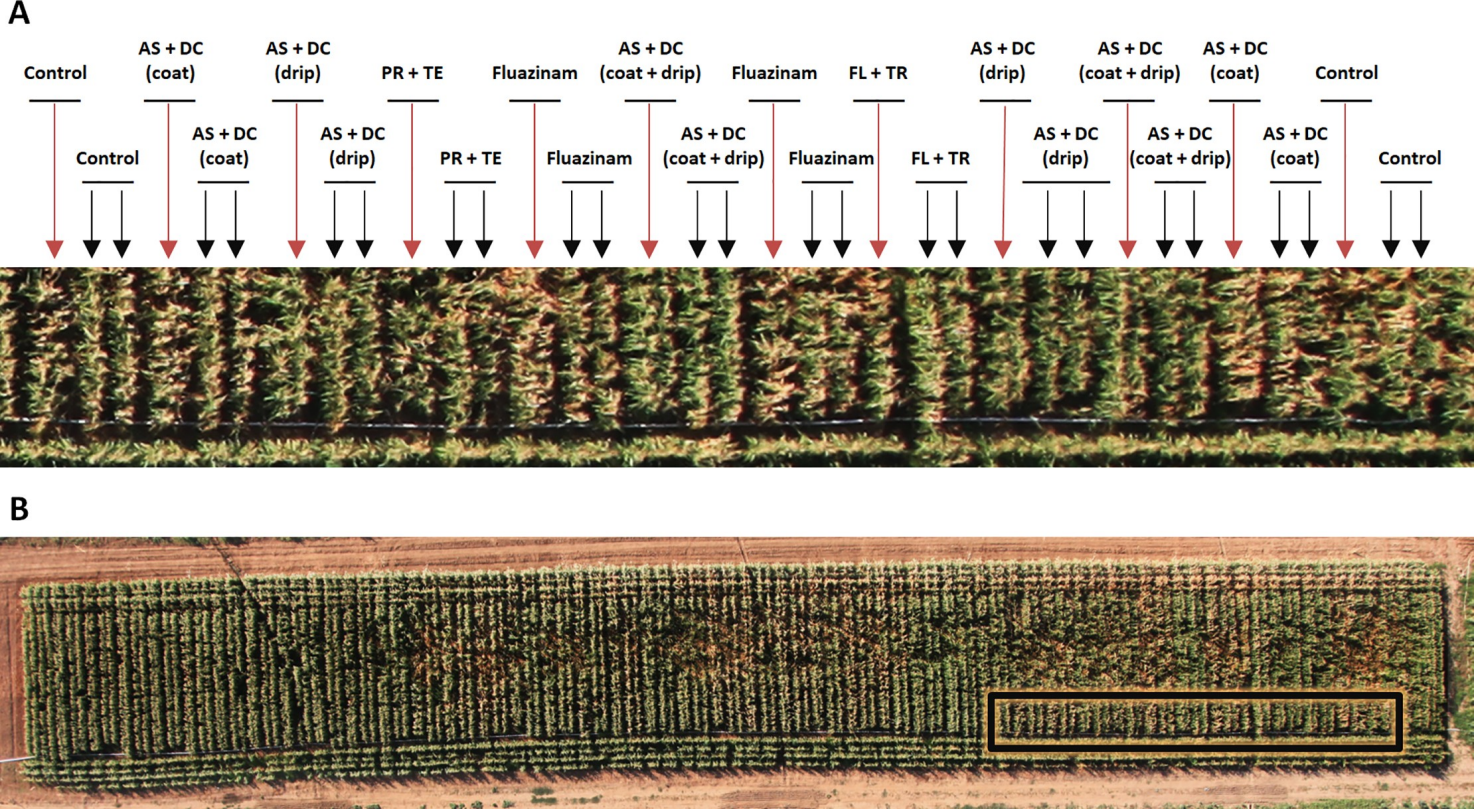
Aerial photograph of the field experiments. The field was photographed 70 DAS (13 DAF). A. Close-up of a portion of the experimental field. B. The whole field in which the portion shown in (A) is marked with a black box. Reddish arrows point to the double-row garden bed (short row spacing of 50 cm) treatments, while the black arrows point to the regular row spacing (96.5 cm) treatments.

The success in restricting the pathogen DNA spread was reflected in the disease symptoms’ severity (Fig 3). Early signs of the disease appeared approximately 50 DAS close to the male flowering (47 DAS). At the age of 70, 23 days after pollination, most of the control (unprotected) Prelude cv. plants were diseased and had dried out (Figs 3–8). In accordance with the literature [4, 11], an increase in drying out upwards occurred, the color of the exterior surface of the lower internodes was altered (Fig 4) and a cross-section of diseased maize plants (Fig 5) displayed a yellow-brown concentration near the first above-ground internode.

Nevertheless, the AS+DC drip protection avoided symptoms in the sensitive Prelude cv. The dehydration appearance (Fig 3), the lower stem symptoms (surface color alternation), the parenchyma tissue wet rot and vascular occlusion (Figs 4 and 5) and the cobs’ symptoms (Fig 6) all clearly demonstrated the influence of the drip protection with AS+DC, which abolished any sign of the disease almost completely. This conclusion supported by the dehydration level measurements (Fig 7), that reveal significant differences between the AS+DC drip protected plants and the control non-protected plants (*P* < 0.05).

At 70 DAS (13 DAF), the AS+DC drip protection inhibited the development of wilt symptoms by 19% in the line-per-row treatment (*P* = 0.05) and by 41% in the double-row treatment (*P* = 0.02). The less pronounced difference from the control in the AS+DC drip protection in the line-per-row treatment is due to a better starting point of the control (25% healthy plants instead of 13% in the double-row cultivation) rather than low effectiveness (both cultivation methods yielded 54–57% healthy plants with a significant reduction in the diseased plant percentages, Fig 7). Indeed it can be noticed that the line-per-row cultivation led to somewhat improved resistance to the disease compared to the double-row cultivation. This effect is probably due to the improved water supply, although all treatments received the same amount of water.

The other treatments, especially the PR+TE and the FL+TR, had a lesser (not significant) but still positive influence in preventing the disease symptoms (Figs 4–8). Based on disease symptoms evaluation alone (70 DAS), the AS+DC seed coating alone and the Fluazinam treatments also had small but evident effects on the symptoms’ outbreak, particularly in the line-per-row treatment.

**Fig 9:**
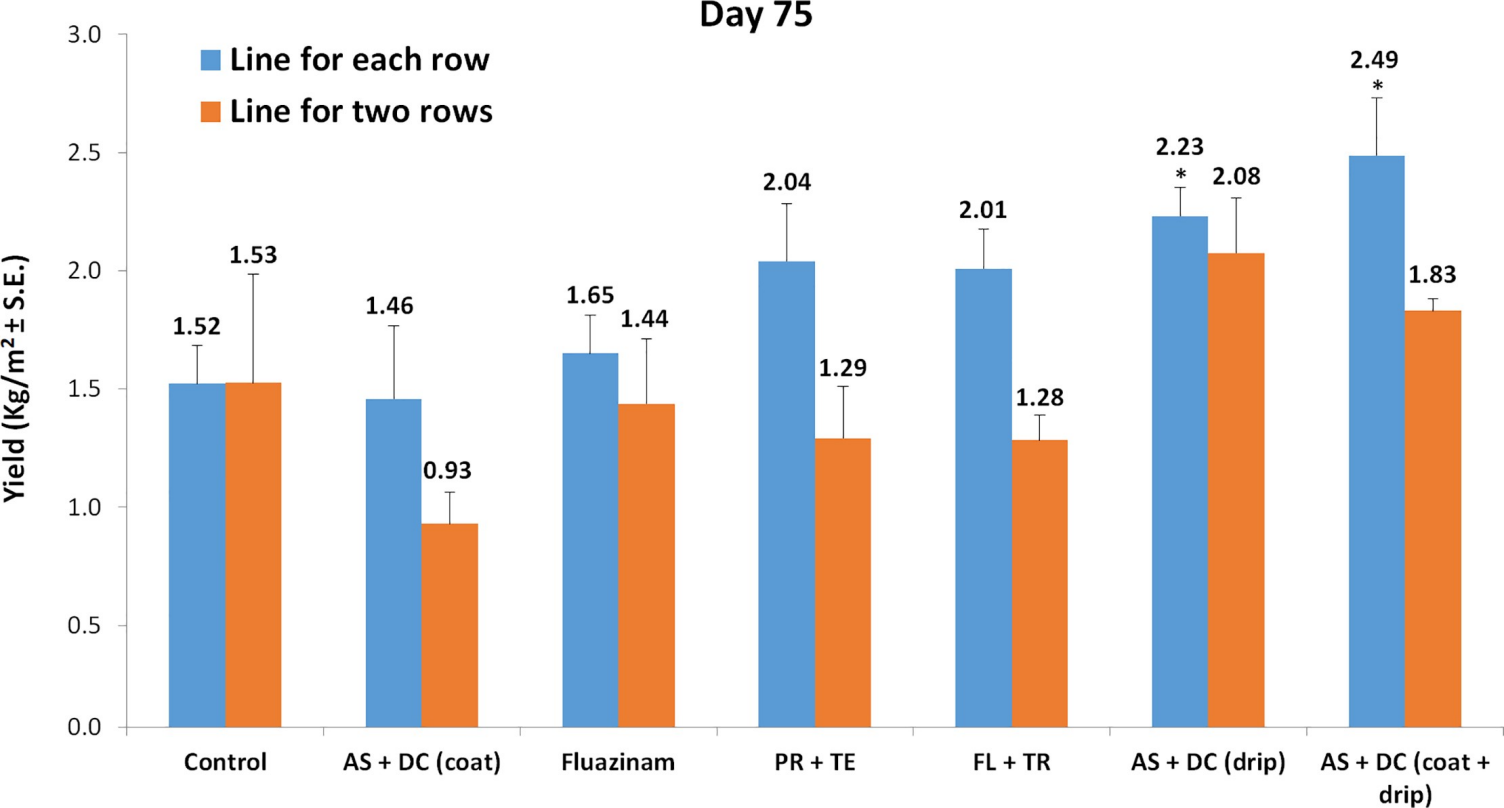
Yield assessment of the field experiments. The assessment was carried out 18 days after fertilization (75 DAS). Values indicate an average of six replications. Error bars indicate standard error. Significance from the control is indicated as * = *P* < 0.05.

As mentioned in the Introduction, maize crop yield of over 20 tons per hectare is considered normal in healthy fields. Here, the untreated controls of both cultivation methods resulted in relatively poor yields of 15 tons per hectare (Fig 9). Remarkably, the most effective treatment, AS+DC drip protection, recovered cob yield by 64% in the line-per-row treatment to 24.9 tons per hectare (*P* = 0.01), and by 36% in the double-row treatment to 20.8 tons per hectare (Fig 9). In the line-per-row treatment, both the PR+TE and FL+TR treatments resulted in crop yield similar to healthy field levels (20.4 and 20.1 tons per hectare, respectively). However, in the double-row growth method, the yields in the PR+TE and FL+TR treatments dropped (as did the overall tendency of all treatments in this cultivation) and reached only 12.9 and 12.8 tons per hectare, respectively.

**Fig 10:**
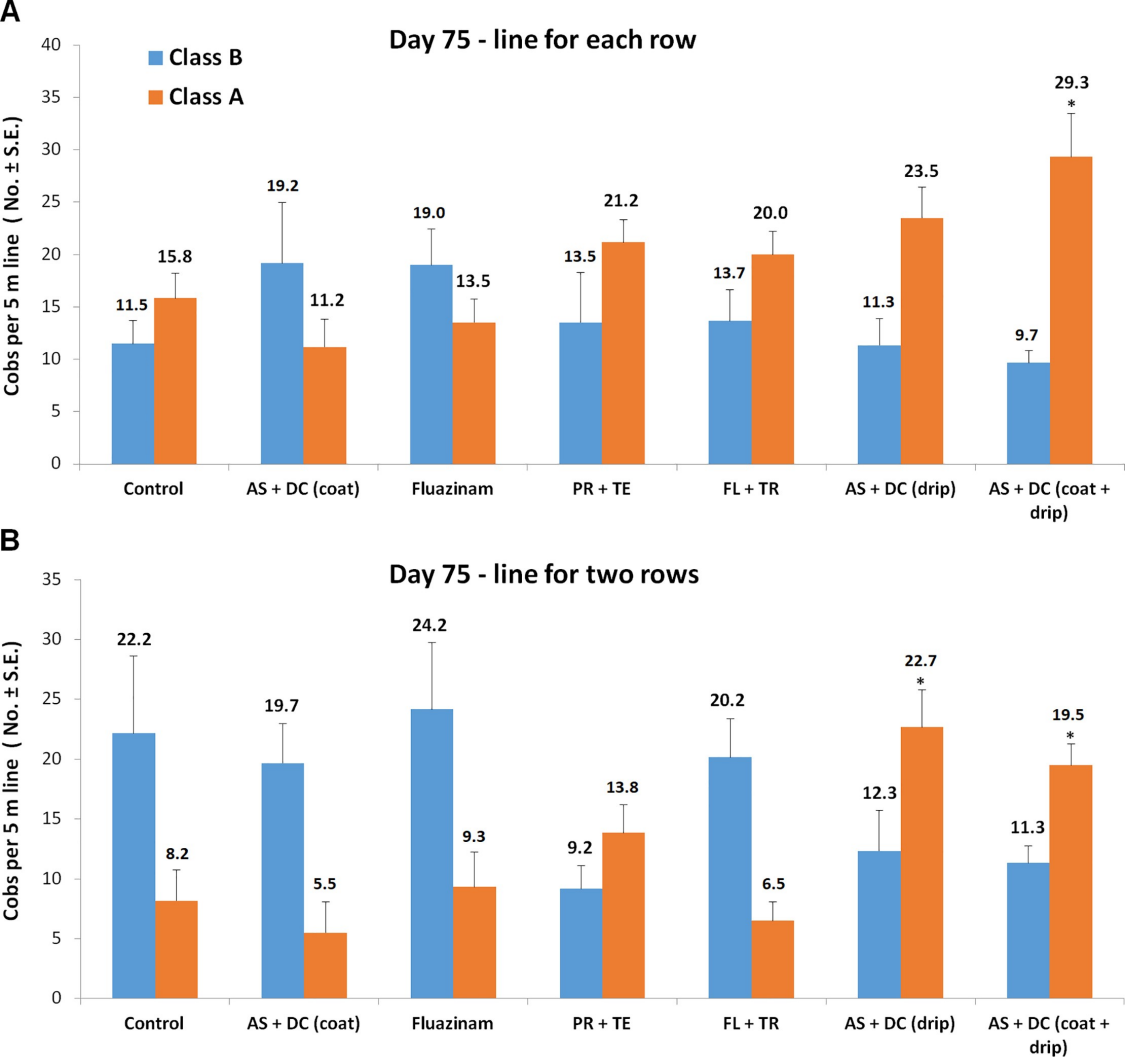
Yield quality of the field experiments assessed 18 days after fertilization (75 DAS). The yield classified as A class had a cob weight exceeding 250 g. Values indicate an average of six replications. Error bars indicate standard error. Significance from the control is indicated as * = *P* < 0.05.

The benefit of AS+DC drip irrigation was reflected not only in cob yield but also in cob quality. The yield classified as A class increased to 75% (1.9 times higher compared to the control) in the line-per-row AS+DC-treated plants, and to 63% (2.4 times higher) in the double-row treatment (significant at *P* = 0.02 and 0.01, respectively, Fig 10). Here, also, in the line-per-row treated plants, the PR+TE and FL+TR drip treatments achieved improved (albeit insignificant) results compared to the control.

## Discussion

*H*. *maydis* causes serious economic losses in maize fields in countries such as Israel, Spain, India and Egypt [4, 6, 11, 32]. Since the restriction of fungicide treatments exerts increasing pressure in many regions, the current control of phytoparasitic fungi tends to rely on cultural practices and host resistance. These procedures are currently used for late wilt damage prevention in Israel. However, *H*. *maydis* is capable of undergoing pathogenic variations [33, 41] and new virulent variants could emerge. Therefore, alternative ways of restraining the disease are continuously being sought.

We had previously established the use of drip irrigation to implement selected fungicides and the chemical seed coating as separate beneficial methods against late wilt in the field [16, 28]. Here, we combined these strategies together with other modifications to achieve high effectivity and relatively more cost-effective treatment to protect sensitive maize hybrids in commercial fields, even in severely infested areas. Indeed, this work is a significant improvement over the successful 2009–2010 experiment [28] in several respects. In this field trial, we examined the use of a drip irrigation line for a double-row garden bed (row spacing was 50 cm) instead of a drip irrigation line for each row (regular row spacing of 96.5 cm). This new method reduces cultivation expenses, human resources and time consumption by nearly half, thus is more cost effective than the 2009–2010 suggested treatment. Due to the shorter distance of the row to the drip line, the coupling-row cultivation enables improved water and fungicide supply (Table 7) compared to the regular two-row cultivation. Moreover, instead of relying on a sole active ingredient, we applied new fungicide mixtures that have a different action mechanism, as detailed in Table 1. These mixtures were injected directly into the drip line according to the successful timetable proven before of three 15-day intervals [28].

The recently developed Real-Time PCR-based assay [16] for tracking *H*. *maydis* infection and spread proved to be a powerful tool for studying the pathogenesis and appraising treatment efficiency (Fig 2). This method is capable of detecting variation in DNA at high levels of 10 HMRT/COX ratios measured at the end of the growth season (70 DAS) in the stem, as well as in small quantities at a scale of about a million times smaller measured in the roots 30 days from sowing (Fig 2).

When quantifying the pathogen DNA using the Real-Time PCR assay, as was done in this study, it is important to note that the correlation between DNA quantity and symptom development has only now started to be revealed. To date, there were several cases when PCR molecular method detected the presence of the pathogen in the host tissues, regardless of the treatment success [28]. Introducing the sensitive and accurate qPCR method [16] is now enabling a better understanding of these connections. Here, the relationship between the observed levels of pathogen DNA in different plant tissues (Fig 2) and plant development stages to ultimate disease incidence (Figs 3–10) was demonstrated. A sharp and exponential tendency of the two measures towards the end of the session was recorded. This is in contrast to a previous report from the same field with the same maize hybrid measured one year earlier [16] of a gradual reduction in DNA inside the host stem towards the harvest day (70 DAS). It was hypothesized that in approaching the end of the growth session when the host tissues dry out, the fungus enters the asexual reproduction stage and develops spores and sclerotial bodies [42], while the primary hyphae biomass gradually comes apart. The results of this work suggest that several factors in the host-environment-pathogen triangle could contribute to the alternative outcome of fungal DNA levels in the host, and that the final status could be different in each experiment.

The fungicides Trifloxystrobin and Tebuconazole are registered for use in maize in Europe and Israel. All other active ingredients presented in this work are not registered for use in maize in Europe and Israel (Data provided by the Israeli Ministry of Agriculture and Rural Development, and by the pesticide companies). This is not surprising since chemical field trials in Israel against the causal agent of maize late wilt were published for the first time only in 2014 [28].

Moreover, the pesticides inspected in the current work were evaluated for the first time against this disease. Thus, there was no justification to register them until now. Also, it should be noted that the use of drip lines to apply pesticides, a method that was rarely used in the past, is becoming more common thanks to the development of low-flow drip irrigation systems and a reduction in drip spacing. This new method is currently being led in Israel by the Netafim Ltd. company. To date, none of the fungicides used in this work are licensed for using the drip irrigation system in Israel. The current work could expedite registration procedures of the antifungal compounds presented here and their use in the drip irrigation system to protect maize. This is especially important since, to date, all other alternative fungicide application methods that were tested in Israel in the field (such as spraying [28]) failed to protect sensitive hybrids.

Combining two or more fungicides with a different mode of action may be crucial in preventing the development of fungal resistance. One example of this threatening risk is Azoxystrobin, a member of the class of quinone-outside (Qo)-inhibiting fungicides (QoIs), which is probably the most successful class of agricultural fungicides [43]. Unfortunately, the rapid development of resistance to this fungicides class and the consequential control failure have become increasingly problematic. Over the past 18 years, more than 30 plant pathogen species distributed across 20 genera were reported to display field resistance toward QoI fungicides [44]. QoI fungicides interrupt mitochondrial respiration by binding to the Qo site of the cytochrome bc1 enzyme complex, impairing electron transfer, and thereby inhibiting mitochondrial respiration and ATP production [43]. Because of their single-site mode of action, QoI fungicides pose a high risk for the revelation of fungicide resistance. Under these circumstances, the suggestion is to avoid using QoI fungicides [45] or to use anti-emergence strategies such as mixing low-risk and high-risk anti-fungal compounds to delay the emergence of resistance to the high-risk fungicide [46].

When comparing the control (unprotected plants) in the double-row cultivation to the control in the line-per-row growth method at 30 DAS (Fig 2), an interesting result was revealed. It can be clearly seen that that infection is reduced under the closer (and thus improved) watering of the line-per-row treatment. Indeed, our experience [28, 35] and a review of the literature support the conclusion that low water potential is one of the most important factors enhancing late wilt disease progression [2, 12, 24, 29]. Nevertheless, the differences during the season between the two cultivation methods were less clear.

Since no other effective strategy is currently available for the integrated management of late wilt in Israel, our study provides an applicative solution to manage this major fungal disease in maize. Future studies should focus on identifying new effective compounds (with a different mode of operation) against *H*. *maydis* and on examining new application methods, such as spraying (with or after scarification soil processing) or using the frontal irrigation system. These application methods are especially important in agricultural fields where the drip irrigation system cannot be applied, or when a combine harvester equipped with a head designed for harvesting rows at a row spacing of 50 cm (S4 Fig), is not available.

## Conclusion

Maize late wilt has been reported to date in more than 10 countries. In the past few years, we examined several chemical applications to restrict *H*. *maydis*, the disease causal agent [16, 28]. This work combined cumulative knowledge with new approaches that rely on changing the cultivation method to two adjacent rows with a drip irrigation line in between, injecting fungicide mixtures into the irrigation drip line in three intervals, and using seeds previously coated with an AS+DC antifungal preparation. One of the treatments, the AS+DC drip protection (alone or in combination with seed coating), was excellent, preventing disease symptoms’ outbreak and restoring yields to a healthy field level. The short row spacing of the double-row cultivation maintained sufficient fungicide concentration in the ground and enabled the chemical treatment success. The AS+DC seed coating treatment alone that was efficient in the early growth stages (up to 50 DAS) provided an additional layer of protection later, which benefited yield production. Today, drip irrigation is considered the most effective chemical means to control maize late wilt disease but also the most expensive of the existing alternatives. The value of drip extensions lines are between 50–60% of total system costs (without installation), so reducing the number of extensions in half could save about 30–40% of the expenses of irrigation, in addition to the savings in manpower and time. As a consequence, 40 years after first becoming evident in Israel [4], we finally have a successful and a more cost-effective treatment that can now be applied in vast areas to protect maize cultivars in commercial infested fields against late wilt.

## Supporting information

S1 Fig A double-row garden bed (row spacing of 50 cm) cultivation method. The photograph of commercial sweet maize field in the Beit She'an Valley, was taken at 30.04.2017.
(TIF)

S2 Fig A six-outlet manifold (Netafim Ltd. Israel) with a specific unit at each outlet was used as a point of injection and mixing of the fungicides with the irrigation water for a homogeneous solution in each different treatment. (TIF)

S3 Fig Soil solution sampling using a MacroRhizon soil moisture sampler (Giesbeek, The Netherlands). Samples were taken approximately 10 cm beneath the ground surface at a distance of 0, 14 and 28 cm from the drip irrigation point.
(TIF)

S4 Fig A combine harvester (owned by Hadasim Agricultural Development LTD) equipped with a head designed for harvesting four rows at a row spacing of 50 cm. (TIF)
