# Supplementary figures and images for: Effective chemical protection against the maize late wilt causal agent, Harpophora maydis, in the field
Source: PLoS One. 2018 Dec 18;13(12):e0208353. doi: 10.1371/journal.pone.0208353 (PMC6298682; doi:10.1371/journal.pone.0208353)

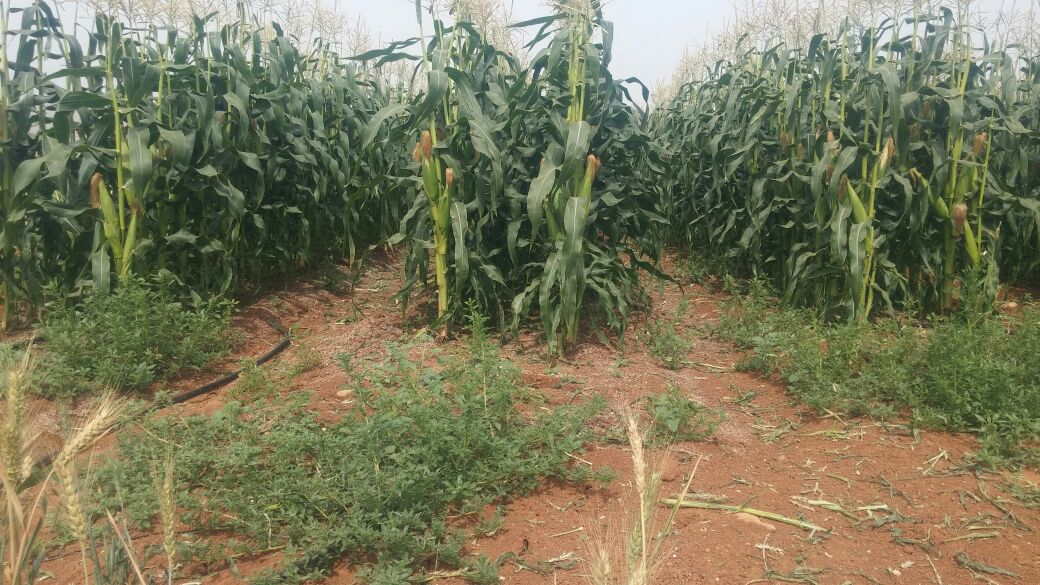

Supplement: S1 Fig — The photograph of commercial sweet maize field in the Beit She'an Valley, was taken at 30.04.2017. (TIF) [file pone.0208353.s001.tif]

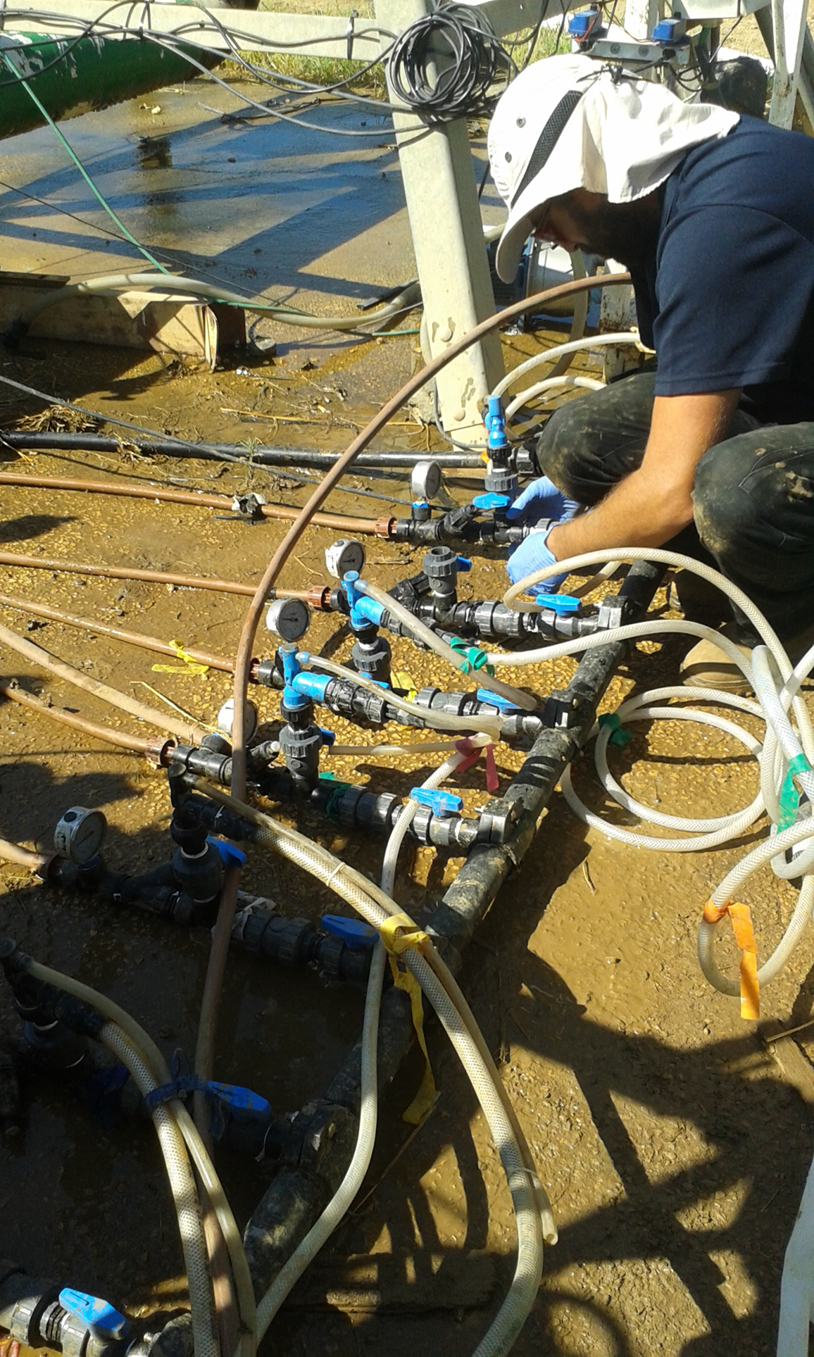

Supplement: S2 Fig — (TIF) [file pone.0208353.s002.tif]

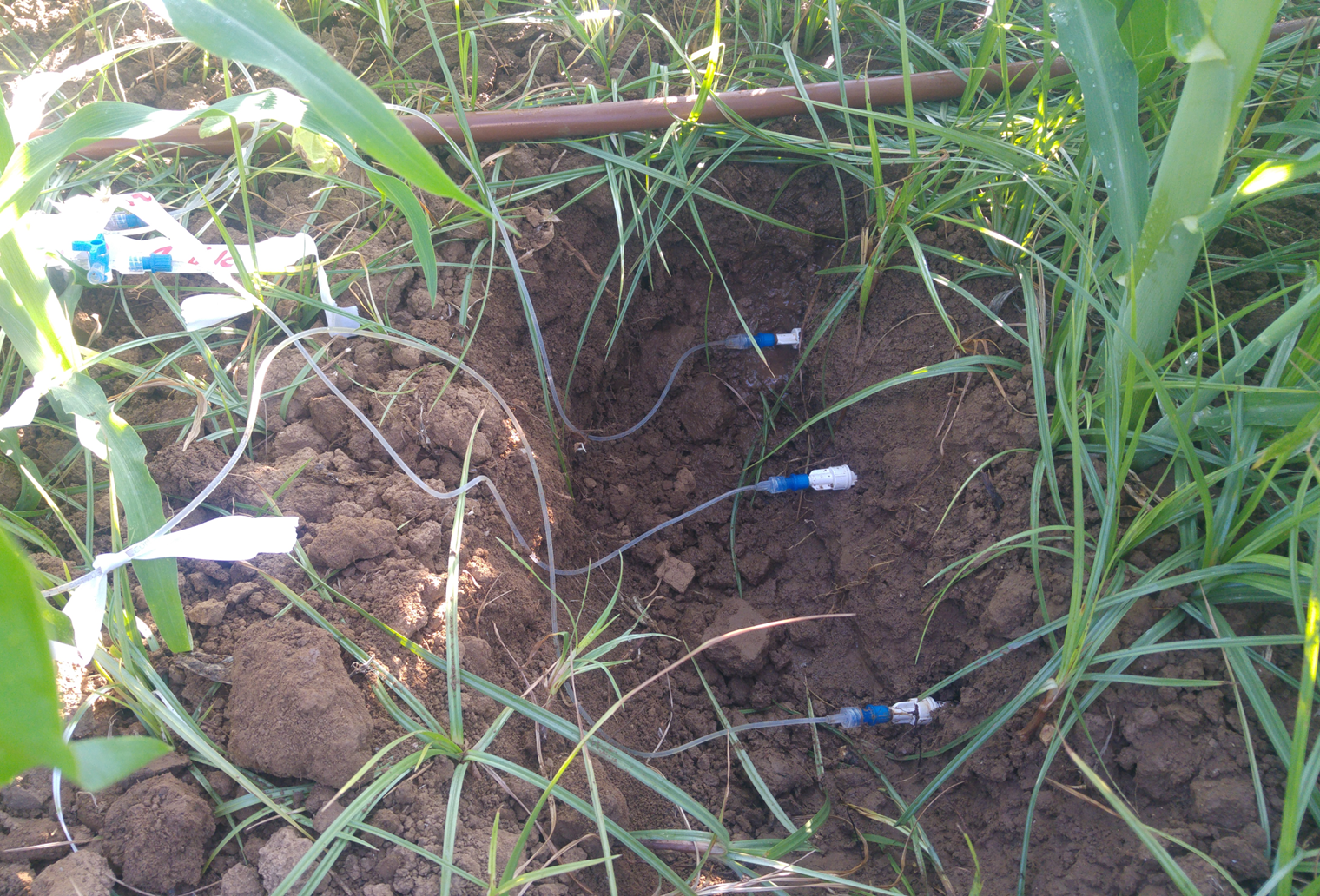

Supplement: S3 Fig — Samples were taken approximately 10 cm beneath the ground surface at a distance of 0, 14 and 28 cm from the drip irrigation point. (TIF) [file pone.0208353.s003.tif]

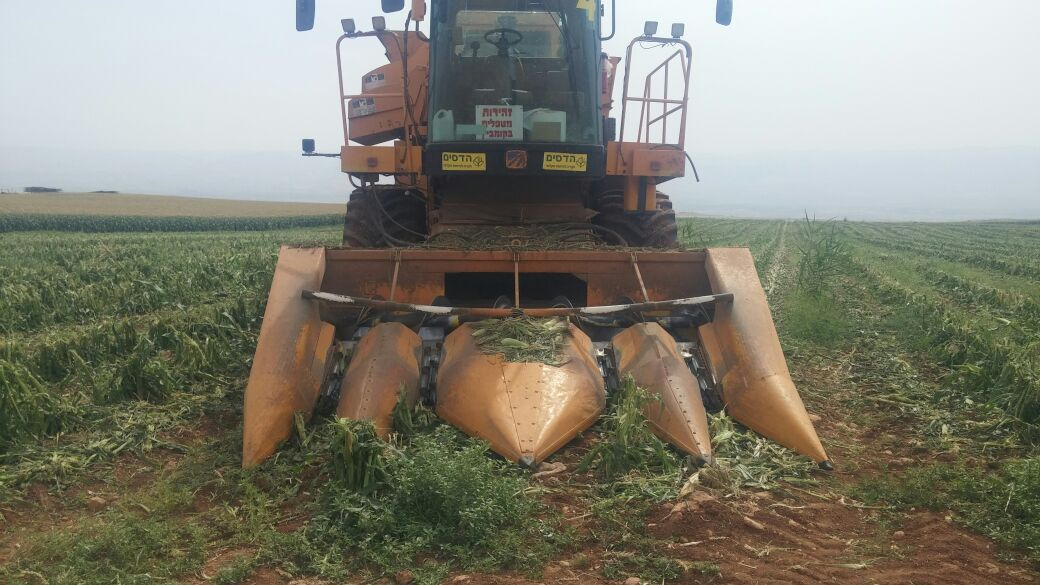

Supplement: S4 Fig — (TIF) [file pone.0208353.s004.tif]
